# Protein Transport through Nanopores Illuminated by Long-Time-Scale Simulations

**DOI:** 10.1021/acsnano.1c01078

**Published:** 2021-06-07

**Authors:** Gregor Mitscha-Baude, Benjamin Stadlbauer, Stefan Howorka, Clemens Heitzinger

**Affiliations:** † Institute of Analysis and Scientific Computing, TU Wien, Vienna, 1040, Austria; ‡ Department of Chemistry, Institute of Structural Molecular Biology, University College London, London, WC1E 6BT, United Kingdom; ¶ School of Mathematical and Statistical Sciences, Arizona State University, Tempe, Arizona 85287, United States; § Institute of Biophysics, Johannes Kepler University Linz, Linz, 4020, Austria

**Keywords:** nanopores, nanoscale-confined space, protein transport, Brownian dynamics, continuum theory, high-throughput simulations

## Abstract

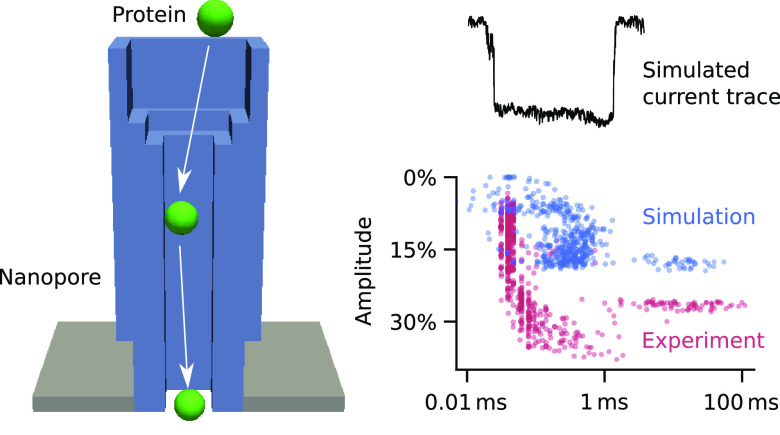

The transport of molecules through nanoscale confined space is relevant in biology, biosensing, and industrial filtration. Microscopically modeling transport through nanopores is required for a fundamental understanding and guiding engineering, but the short duration and low replica number of existing simulation approaches limit statistically relevant insight. Here we explore protein transport in nanopores with a high-throughput computational method that realistically simulates hundreds of up to seconds-long protein trajectories by combining Brownian dynamics and continuum simulation and integrating both driving forces of electroosmosis and electrophoresis. Ionic current traces are computed to enable experimental comparison. By examining three biological and synthetic nanopores, our study answers questions about the kinetics and mechanism of protein transport and additionally reveals insight that is inaccessible from experiments yet relevant for pore design. The discovery of extremely frequent unhindered passage can guide the improvement of biosensor pores to enhance desired biomolecular recognition by pore-tethered receptors. Similarly, experimentally invisible nontarget adsorption to pore walls highlights how to improve recently developed DNA nanopores. Our work can be expanded to pressure-driven flow to model industrial nanofiltration processes.

The transport of molecular matter across membrane pores is of relevance in biology, industry, biotechnology, and research. Biological pores help shuttle small-molecule cargo or proteins across bilayer membranes to maintain cell function. In industry, synthetic porous membranes enable filtering for desalination,^1−4^ oil processing,^5^ gas separation,^6^ battery regeneration,^7^ biopurification,^8^ and blood dialysis,^9^ by acting as selective barriers that permit pore passage for one or a few molecular species while rejecting others. By comparison, in biotechnology and research, single nanopores enable DNA sequencing and sensing of individual biomolecules^10−14^ that pass the nanopore one at a time.

Understanding the principles underpinning transport of biomacromolecules is required to explain biological nanopores, filtration, and sensing and to rationally design nanopores. Transport selectivity is usually based on size exclusion, and hence nanopore geometry and dimensions,^8,15^ but also on molecular recognition *via* cognate receptors present inside the pore. Transport is also influenced by electrostatics and hydrodynamics, which can vary within the channel lumen.^16−22^ Nanoscale transport is best studied with resistive-pulse sensing, where individual molecules passing through the nanopore are registered *via* temporal changes of a transmembrane ion current, as used in DNA sequencing^10,11,13^ and single-molecule protein sensing.^12,23^ Yet, the experiments do not offer a dynamic picture of the detailed transport processes, leaving several key questions unanswered: What is the trajectory of a protein entering a channel and what is the probability that the molecule binds to a recognition site rather than simply passing the nanopore? Furthermore, does binding to a recognition site follow the strength expected from solution studies, and what is the extent and nature of nonspecific binding to a channel wall?

Simulations can be a computational microscope to explain experimental observations and inform rational design. Protein transport through nanopores has been simulated using all-atom molecular dynamics (MD),^24−26^ Brownian dynamics,^27−30^ and continuum theory^31,32^ with the first route offering the highest level of molecular detail.^33^ Yet, fundamental insight is limited by the computational difficulty of (i) examining transport events in the milliseconds-to-seconds range typical for protein translocation^34−36^ and (ii) simulating hundreds of events required to draw statistically valid conclusions. One approach to bypass long time scales is steered MD, where a molecule is forced to translocate with artificially high speed in nanoseconds.^24,25,37^ But this does not yield the true distribution of event durations and may also distort the description of physical processes.

To promote a fundamental understanding of nanopore transport, this study describes a coarse-grained, high-throughput computational approach to generate hundreds of millisecond-to-second protein trajectories under experimentally realistic conditions to answer all scientific questions mentioned above. The computational approach combines Brownian dynamics (BD) with continuum simulations based on the Poisson–Nernst–Planck–Stokes (PNPS) equations (Figure 1A). Proteins are modeled as beads lacking internal conformations to enable fast computations. BD simulations calculate the trajectories using the Langevin equation^27−30^ to determine particle motion from stochastic forces such as collisions with the solvent molecules, in combination with a deterministic driving force. In comparison, the PNPS equations determine the ion current as well as the electrophoretic and electroosmotic driving forces acting on the protein at any particular position, depending on factors such as geometry, applied voltage, partial charges, and ion concentration. We decouple continuum calculations from BD simulations and compute forces *a priori* for a fixed number of protein positions, relying on a 3D finite-element code for PNPS.^38^ The resulting force field is used to simulate protein trajectories where the current read-out depends on the exact position of the protein inside the nanopore.

**Figure 1:**
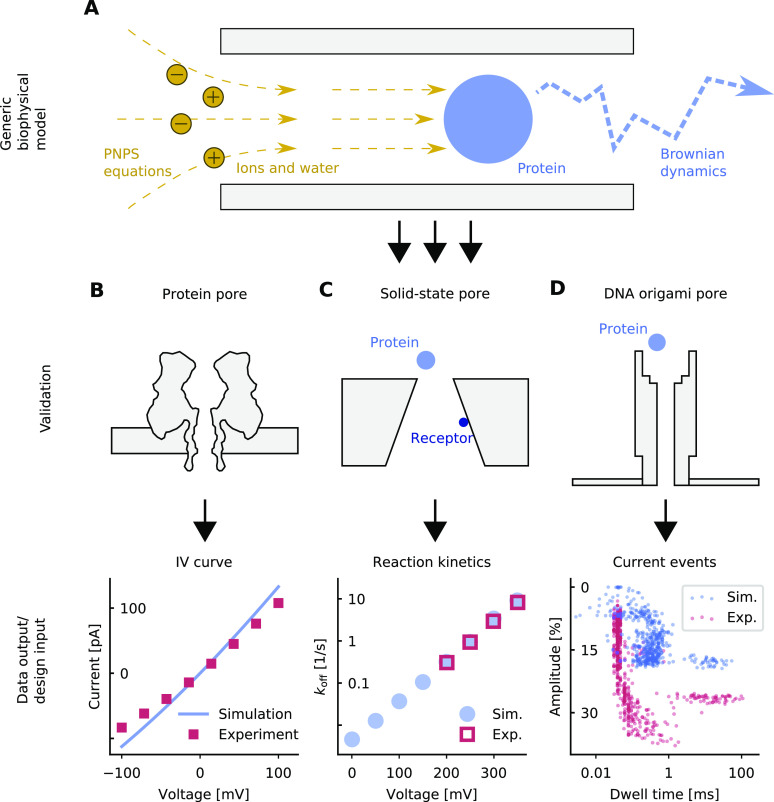
Modeling nanopore transport. (A) Sketch of a biophysical model which combines the PNPS equations as a continuum framework with BD simulations for a molecule’s trajectory. (B–D) The model is applied to different nanopores to compare the simulated transport of ions and proteins with experimental measurements.

We use three representative nanopores to illustrate that our modeling approach advances the field. Using the reference protein pore α-hemolysin (Figure 1B) we demonstrate how the simulations take full account of the position dependence and anisotropy of diffusion coefficients for translocating molecules in order to calculate their trajectories. Unlike previous studies^39−44^ we model the diffusivity of ions and proteins as a function of radial and axial position using a mix of numerical computation and analytical approximation, thereby achieving a good match to experiments, independent of any fitting parameter (Figure 1B).

Using a solid-state nanopore carrying a chemical receptor for a protein^35^ (Figure 1C), we also model protein transport and specific binding. Stochastic Poisson-guided binding and optional rebinding^45^ occurs to a recognition site inside the pore when the protein stays within a binding radius. The binding durations depend on the force acting on the protein. These simulations answer questions on the probability that the protein binds within rather than passes through the pore. The simulations also compare binding strength in pores *vs* solution.

Finally, we apply our model to a DNA nanopore to simulate nonspecific protein–pore interactions, calculate current blockade traces, and compare them to experiments (Figure 1D). Nonspecific interaction results from complicated arrangements of covalent, ionic, hydrogen, and van der Waals bonds^34,45^ and involves an energy barrier as the dwell times exponentially depend on applied voltage.^34^ To account for nonspecific binding, we adapt the targeted binding model by expanding the adsorption area to the entire pore wall. Through exploratory comparison of our simulated and the experimental current traces, we are able to narrow down the location of the prospective binding sites within the nanopore in the absence of any detailed structure.

## Results and Discussion

### Modeling Ion Diffusivity in α-Hemolysin

**Figure 2:**
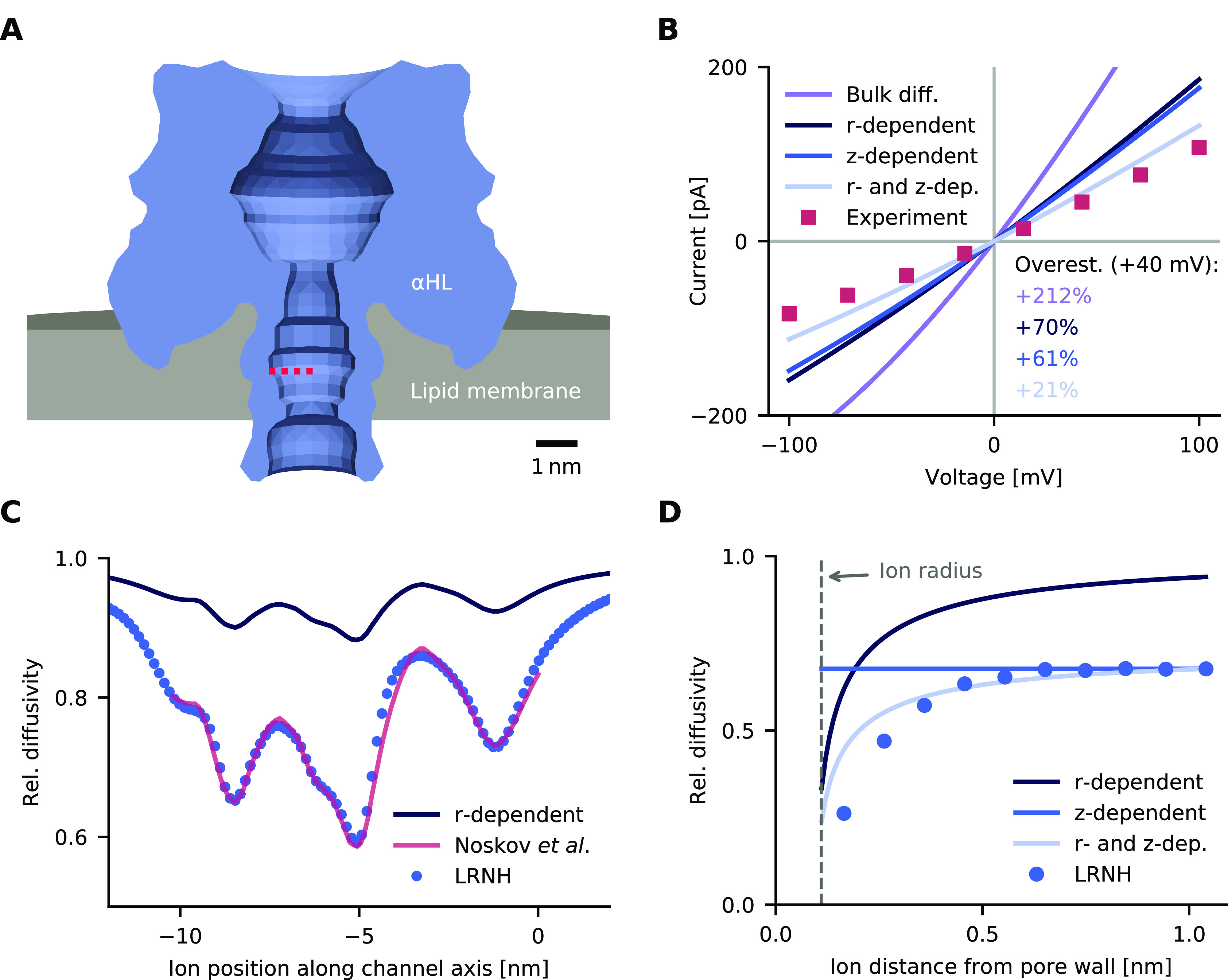
Ion currents across α-hemolysin. (A) Finite-element model of the protein channel. (B) *IV* curves obtained from the different diffusivity models and experimental values measured in 1 M KCl.^46^ (C) Position-dependent ion diffusivity at different positions along the central channel axis. The values represent the *z*, *z* component of the 3 × 3 diffusivity tensor. (D) Ion diffusivity at different pore radii along a channel cross-section, which is indicated in panel A by a dotted red line.

To demonstrate the ability of the approach to predict position-dependent ion diffusivity, we calculated ion currents through the α-hemolysin pore (Figure 2A) and compared them to experimental^46^ current–voltage curves (Figure 2B). Exploiting well-defined structure of the pores, an axisymmetric geometric pore model was built (Supplementary Section S1), and diffusivities under various assumptions were calculated (Supplementary Section S2).

To simulate ion currents, we solve our continuum model under different applied voltages using finite elements. Briefly, a simulation consists of solving differential equations (PNPS) describing the interaction of the electrostatic potential, anion and cation concentrations, fluid velocity, and pressure on a computational domain that includes the pore, membrane, and surrounding fluid reservoir. Details can be found in the Methods.

A crucial input to realistic simulations is the diffusivity of ions. Our baseline model for ion diffusivity is low-Reynolds-number hydrodynamics (LRNH), which yields position-dependent diffusivities in a parameter-free way.^47−53^ LRNH captures experiments well in channels with diameters greater than 1 μm.^54^ We computed LRNH diffusivities at different positions along the central pore axis (Figure 2C, blue circles) and at different radii along a channel cross-section (Figure 2D, blue circles; the cross-section is indicated in Figure 2A, dotted red line). The diffusivities in these figures are relative to the bulk diffusivity, which corresponds to a value of 1.0.

Applying the LRNH model to our continuum simulation of ions is too costly from a computational point of view, especially in our later setup, where ion diffusivity is coupled to the protein position. Thus, we are seeking an efficient approximation of LRNH. One approach is to ignore the radial variation and extend the computed values at the pore center (Figure 2C, medium blue circles) to the rest of the pore, yielding a purely *z*-dependent diffusivity (Figure 2D, medium blue line). This was first used by Noskov *etal*.,^55^ who did not have LRNH calculations at hand but used tabulated values for LRNH in an infinite cylinder with a radius equal to the *z*-dependent pore radius, which comes close to the full model (Figure 2C, red line).

But a purely *z*-dependent model is not satisfactory: as we see in Figure 2D (LRNH), diffusivity is reduced much stronger near the channel walls. On this length scale, water is a highly viscuous fluid that acts like glue between the static wall and moving ion.^47,56,57^ To account for this, we propose an *r*-dependent diffusivity model that depends solely on the distance to the nearest wall. Given the distance *r*, we take the diffusivity that would arise if the particle were located at distance *r* from an infinite plane wall. For this situation, approximate closed-form solutions exist.^47^ The *r*-dependent profile is of similar shape to a profile derived from MD simulations^57,58^ with ions confined on one side; see Supplementary Figure S2a.

On its own, the *r*-dependent model generally overestimates diffusivity, because walls on all sides reduce diffusivity more strongly than one plane wall does (Figure 2C and D, dark blue line). But if we normalize the *r*-dependent model by a *z*-dependent factor, such that it equals the LRNH result at the channel center, we get a combined *r*- and *z*-dependent model that captures the full LRNH model near the channel walls quite well (Figure 2D, light blue line). This approximation slips when the channel diameter is comparable to the size of the diffusing particle (Supplementary Figure S2b).

Our model agrees well with MD calculations by Bhattacharya *etal*.,^46^ who report an average diffusivity of K^+^ ions in α-hemolysin of 0.56 times the bulk value. In our model, the average relative diffusivity is 0.47. It is computed by averaging our *r*- and *z*-dependent model over the channel, weighted by the simulated K^+^ distribution.

When comparing *IV* curves for different diffusivites, we see that the simplest assumption—no position-dependence—leads to a gross overestimation of the conductance by 212% (Figure 2B, purple line). Both the *z*-dependent (+61%) and the *r*-dependent model (+70%) reduce overestimation considerably (Figure 2B). By far the most realistic result is attained by the combined *r*- and *z*-dependent model, with a conductance of 1.28 nS at +40 mV, comparable to the 1.05 nS reported in experiments^46^ (+21%).

### Analyte Binding in a Receptor-Modified Solid-State Pore

**Figure 3:**
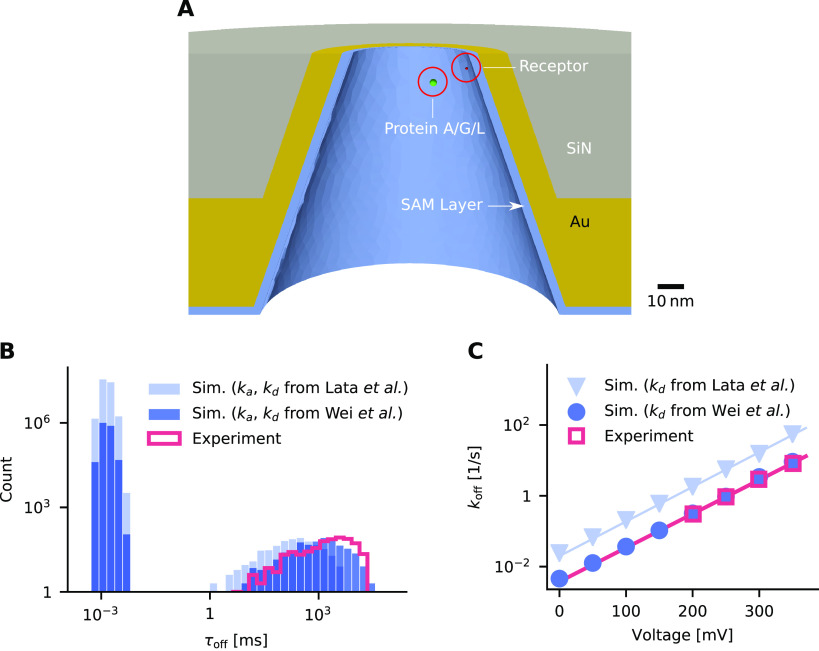
Protein binding to a receptor within a solid-state nanopore. (A) Finite-element model of the nanopore and its material composition. (B) Histogram of simulated and experimental binding event durations. The rate constants *k*_a_ and *k*_d_ used for simulating binding were taken from ref (59) (light blue bars) or inferred from measurements of ref (35) (dark blue bars). The experimental τ_off_ data (red line) are from ref (35). The transmembrane voltage is 200 mV. (C) Calculated and experimental values for rate constant *k*_off_ at different voltages. The color code is as in panel B. Straight lines are least-squares fits to symbols of the same color.

After validating our calculations of diffusivity, we applied the computational approach to protein transport to test the validity of our binding model and clarify the following questions: Which percentage of trajectories leads to binding onto the target site? And can measurements of kinetic reaction rates be transferred from solution to a nanopore? We answered the questions with the example of a solid-state nanopore and molecular recognition data by Wei *etal*.^35^ The conically shaped SiN solid-state nanopore is coated with a self-assembled monolayer of protein-repelling polymer chains (Figure 3A, SAM) and carries an anchored single NTA_2_ receptor capable of recognizing His_6_-tagged protein A/G/L analyte (Figure 3A). The strength of the NTA_2_–His_6_ interaction is described by the duration τ_off_ of the individual binding events within the nanopore. The binding is stochastic, and hence duration values are exponentially distributed (Figure 3A, red line in histogram). Complementary binding data come from ensemble solution measurements by Lata *etal*.^59^ providing association rate constant *k*_a_ and dissociation rate *k*_d_. In these measurements, His_6_ and NTA_2_ bind and dissolve in free solution and are both coupled to fluorescein to make these interactions visible. We expected that the dissociation rate would transfer well to the nanopore context and simulations would thus reproduce experimental τ_off_ observations when using this dissociation rate in the binding model.

We built a model of the nanopore and simulated translocation of the protein A/G/L analyte and its binding to tethered NTA_2_ (Supplementary Section S3). The protein is represented as a bead of 3 nm radius with a net charge of −50*q*. We first calculated forces on the protein with the PNPS equations (more details below). Protein trajectories were computed by placing the protein at the bottom of the pore and evolving its position with Brownian dynamics. In a third step, we analyzed the amount of time trajectories spent within the binding zone of the receptor and calculated stochastic binding events whose duration was included in the protein dwell times. When the protein was near the receptor, ion current was reduced by 0.5%, similar to experiments where the blockades range from 0.5–1%.^35^

Analyte binding was calculated with the solution-based reaction rates *k*_a_ and *k*_d_ of Lata *etal*. and, in a second set of simulations, with different rates that were inferred directly from nanopore measurements of Wei *etal*. in a slightly larger but similar pore. The applied transmembrane voltage was set at −200 mV. The computed protein dwell times are shown in Figure 3B. Analysis of more than 10 million protein trajectories revealed that at least 99.97% of the events did not lead to the targeted binding. Furthermore, the event durations were short (1–10 μs). These events are undetected in experiments, and the sheer scale is striking in comparison to the miniscule fraction of detected binding events.

Further analysis revealed that binding between protein and receptor is likely underestimated when using the association rate constant from solution measurements. With *k*_a_ from Lata *etal*., only 0.0008*%* of the simulated events lead to binding (Figure 3B, light blue bars). A more realistic estimate for the percentage of binding events can be obtained by dividing the experimental event rate from Wei *etal*. () by the theoretical rate of arrivals at the pore;^60^ see Supplementary Section S3 for details. According to this estimate, 0.027% of events lead to binding, which is 34× higher than the 0.0008% computed with *k*_a_ from Lata *etal*. To account for the difference, in Figure 3B (dark blue bars) we use a 34× larger *k*_a_ (5.2 × 10^6^*vs* 1.5 × 10^5^ M^–1^ s^–1^). Such an increase in association is plausible given the reduced diffusivity of protein A/G/L and the surface-anchored receptor compared to the small molecules used in solution measurements,^59^ as well as the favorable rotation angle of the receptor.^61,62^

Protein trajectories with receptor binding were longer (10 ms to 3 s) than the nonbinding events, and the inferred event duration was in histogram analysis close to the experimental data from Wei *etal*. (Figure 3B, data, red; simulation, dark blue bars). A small discrepancy between simulated and experimental durations (Figure 3B and C) was found when using the dissociation rate from Lata *etal*. (Figure 3B and C; simulation, light blue bars and triangles). Dissociation seems to occur more slowly when the protein is attached to the nanopore. This could point to an additional (not present in our model), weaker interaction between the attached protein and the pore surface and/or a kinetic confinement of the reacting His_6_ part, which hinders dissociation.

Similar agreements were found when simulations were run at transmembrane voltages different from −200 mV (Figure 3C). For this analysis, the mean event duration  was converted into the rate  as in ref (35). The voltage dependence of simulated *k*_off_ had the same slope as in experiments, but the voltage range was larger in the simulations (Figure 3C; simulation, blue circles; data, red squares). Our results from simulating the solid-state pore validate the applicability of our binding model to protein diffusion and recognition inside nanopores.

### DNA Origami Nanopore

**Figure 4:**
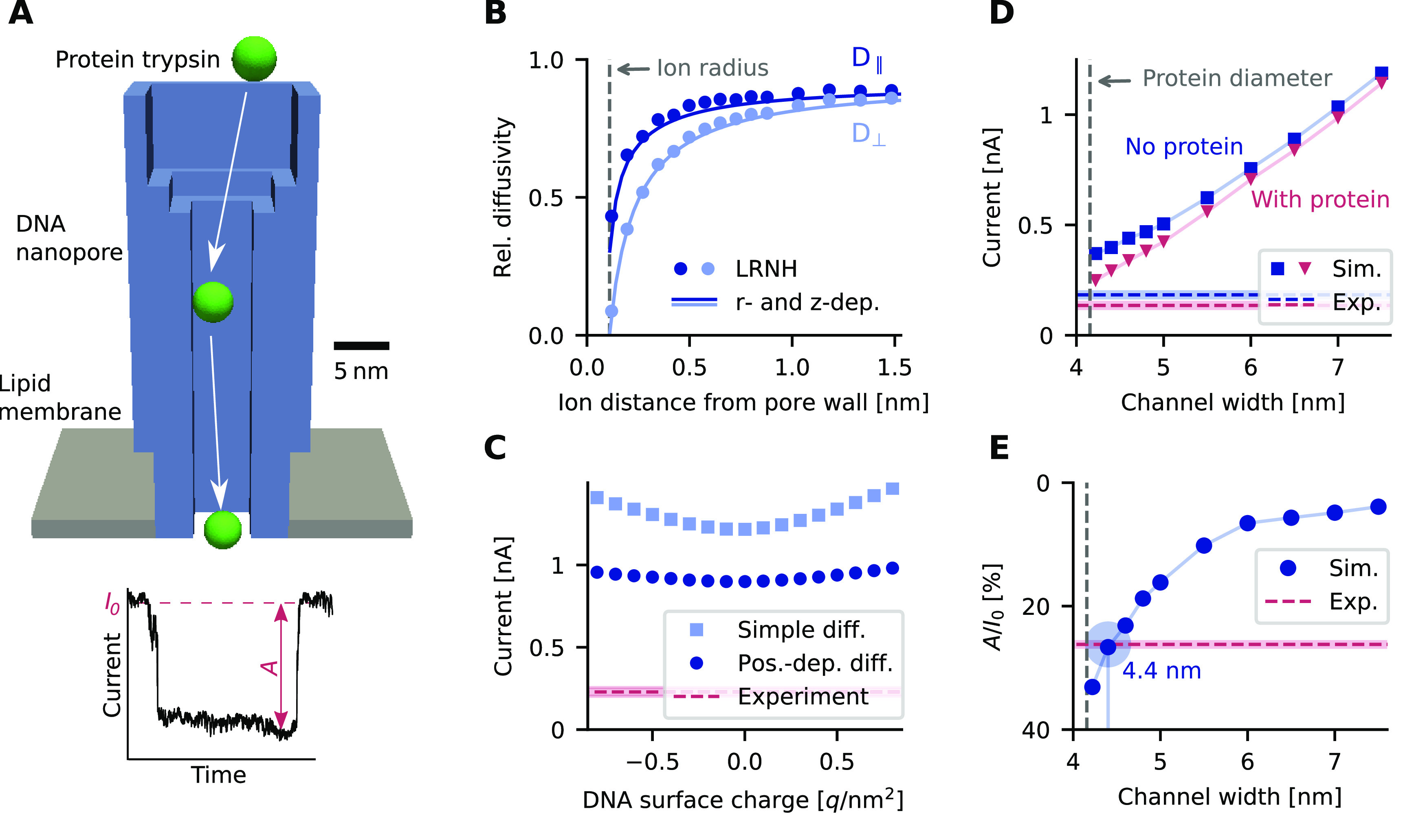
Ion diffusivity and ion currents in a DNA nanopore. (A) Model of a DNA origami nanopore from ref (36) and a schematic current trace. (B) Tangential (D_∥_) and normal diffusivity (D_⊥_) of K^+^ ions in the channel for different diffusivity models. (C) Simulated ion current as a function of DNA surface charge and diffusivity. Dark blue circles: position-dependent diffusivity model used elsewhere in the paper. Light blue squares: simpler, piece-wise constant model where diffusivity takes a constant value in the pore computed for the pore center and another constant value in the bulk. Dashed lines in this and panels D and E are the experimental values from recordings using a pore described in ref (36); shaded areas indicate the variation in measurements. The voltage bias is −100 mV and the electrolyte is 1 M KCl. (D) Simulated ion current as a function of channel width, with (red triangles) and without (blue squares) protein at the pore center. The corresponding measurements are indicated by a dashed line. The voltage bias is −80 mV at 1 M KCl. (E) Relative current blockade with a protein in the pore lumen, as computed from panel D.

After validating our simulation model for specific protein pore interaction, we explored nonspecific protein adsorption at the example of a DNA origami nanopore^36^ by simulating current traces of the blockade events and comparing them to experimental data (Figure 4A). The walls of the origami pore are composed of up to three layers of interconnected DNA duplexes that enclose a 46-nm-high channel lumen of a nominally 6 × 6 nm^2^ cross-section (Figure 4A). The detailed structure of the recently developed class of DNA nanopores is more dynamic than that of protein pores due to the inherent flexible bending of DNA duplexes.^63−65^ Furthermore, the precise shape and dimensions of the bilayer-spanning pore section may deviate from the nominal square due to lateral membrane pressure. Simulating ionic properties for this DNA nanopore is hence a challenge but also a test bed for our modeling route. The DNA nanopore is also ideal to explore protein translocations with intermittent nonspecific binding to the pore wall. The average conductance of the pore has been reported^36^ as well as translocation traces for trypsin including unusually long events that probably arise from nonspecific binding (Figure 4A). The aim of the simulations was to model the uncertain shape and explain how nonspecific interaction of proteins to the pore can account for long events.

We modeled the pore as a layered structure (Figure 4A) and approximated trypsin as a charged sphere of radius 2.08 nm, corresponding to its crystallographic dimensions of 4.3 × 3.8 × 2.3 nm^3^ and its isoelectric point of 10.1. The interactions of the DNA pore with ions was examined first. As shown in Figure 4B, the diffusivity of K^+^ is strongly reduced close to the pore walls, in agreement with the model’s no-slip condition. Compared to α-hemolysin, our *r*- and *z*-dependent approximation matches the LRNH model very closely in Figure 4B, due to the larger diameter of the DNA pore.

We next examined how the surface charges on the pore walls influence the ion current through the pore. The surface-charge density of DNA is usually estimated as −0.74 *q*/nm^2^. The density was varied in our calculations from −0.8 to 0.8 *q*/nm^2^. The simulated current considerably changes with surface charge density (Figure 4C, light blue squares) under the simplifying assumption of a constant ion diffusivity inside the pore. Using the more realistic and previously established position-dependent diffusivity caused a predictable drop in current; yet the influence of the surface charge was almost negligible (Figure 4C, dark blue circles). Regardless of parameter settings, the simulated current was 4–7 times higher than the experimental current of 229 pA at −100 mV (Figure 4C, red dashed line). By comparison, MD simulations of conductance in similar pores agree well with experiment.^65^

To address the conductance difference, we accounted for the known structural dynamics of the pore^64^ and explored how varying the channel width alters the ion current. The simulations reveal that only narrow channel widths of around 4 nm yield currents (Figure 4D, squares and triangles) close to the experimental conductance (Figure 4D, dashed lines). Additional insight can be gained by comparing the relative current blockade. The blockade is defined as *A*/*I*_0_ where *I*_0_ is the open pore current and *A* the difference between open and blocked pore current (Figure 4A). Agreement with the experimental blockade value of 26.2 ± 0.7% was observed with a modeled channel width of 4.4 nm (Figure 4E). When assuming a circular instead of square cross-section of the channel lumen, experiments are matched at a width of 4.9 nm (Supplementary Figure S5).

We stress that the mismatch in relative blockades cannot be rationalized by a reduced ion diffusivity or ion–DNA binding, as these would affect open and blocked pore currents in a similar way. Thus, while a significant reduction of the nominal channel width of 6 nm is not supported by MD simulations,^65^ our results still suggest phenomena that effectively reduce the available space for ions. In particular, continuum simulations should incorporate finite ion size and discrete charge effects.^66−68^

### Simulation of Protein Traces

Next, we simulated protein movement through the nanopores with an unaltered channel width of 6 nm. To set up the calculation of trajectories, we created a grid of protein positions that covers the channel and fluid reservoir. For each grid position *x*_*i*_, we solved the PNPS equations with the protein centered at *x*_*i*_ and computed the force *F*(*x*_*i*_) and current *J*(*x*_*i*_). Interpolation of these grid values allows fast evaluation of *F*(*x*) and *J*(*x*) for arbitrary positions *x* during the BD simulation, where the force drives protein trajectories.

**Figure 5:**
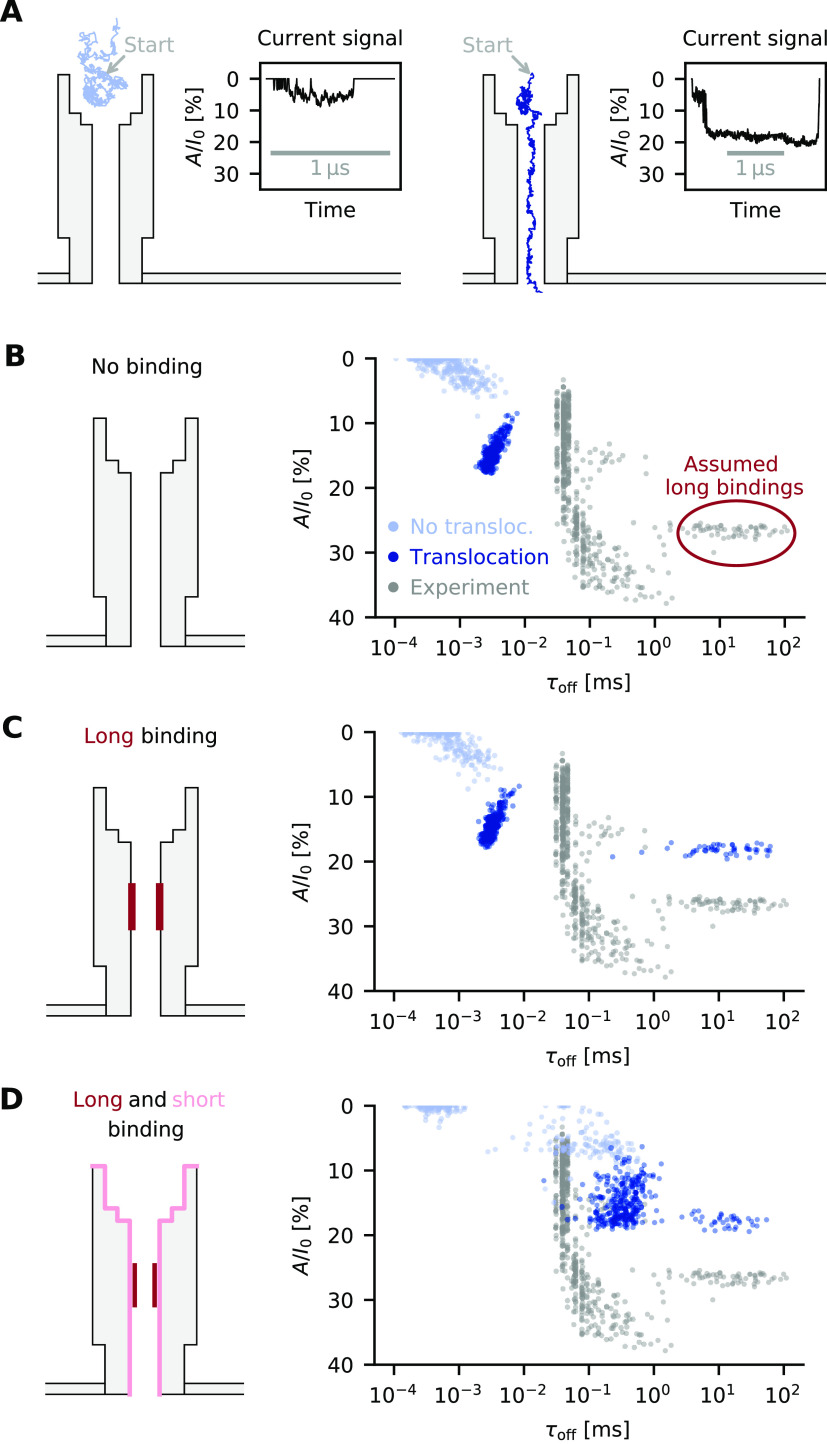
Transport of protein trypsin across the lumen of the DNA nanopore. (A) Simulated trajectories (light and dark blue line) and associated current traces, where the protein either failed to translocate the pore (left) or successfully entered and translocated (right). (B) Scatter plot of events with duration τ_off_ and normalized amplitude *A*/*I*_0_. Colored dots represent simulated events (light blue: failed translocation, dark blue: successful translocation); gray dots are experimental data from recordings using a pore described in ref (36). (C) Event scatter plot with long binding in the middle of the pore. (D) Event scatter plot with long binding in the center of the pore and short binding along the entire pore wall.

At the start of a BD simulation, the protein was placed directly at the wider entry of the nanopore (Figure 5A). The trajectories stopped if either (a) the protein successfully translocated to the lower end of the nanopore or (b) the protein diffused in the wrong direction leaving a defined boundary box of 10 × 10 × 12 nm^3^ above the upper entry. The trajectories and evaluation of *J*(*x*) were used to calculate the current blockade traces. Two representative read-outs for successful and nonsuccessful translocation are shown in Figure 5A.

The simulations were carried out for over 500 trajectories at −80 mV assuming that electrophoresis and electroosmosis are driving translocation; protein binding to the pore wall was excluded. The simulation results are summarized in Figure 5B in a scatter plot where each event is presented as a dot with a defined simulation time τ_off_ and normalized amplitude *A*/*I*_0_. The events cluster into two regions. Nontranslocations (Figure 5B, light blue) were clustered between 0.1 and 1 μs event duration. By comparison, successful transport events (Figure 5B, dark blue) were longer at 1–10 μs, in line with the slower process of translocation. This event class also had a more extensive current blockade, as expected when the protein blocks the pore during translocation.

By comparison, the experimental data (Figure 5B, gray dots) had longer durations mostly in the range of 30 to 300 μs and a blockade level ranging from 5% to 35%. They also featured a separate cluster with very long event durations from 1 to 100 ms (Figure 5B, encircled). Translocation events faster than 30 μs were not detected in the experiments due to the inherent electrical filtering.^60^ The up to 10 000-fold difference in simulated and experimental durations suggests that electrophoresis/electroosmosis alone cannot account for translocation.

We set out to explain the cluster of very long events by considering that they stem from the binding of protein to the pore wall (Figure 5C). The binding was assumed to occur to a defined pore region (Figure 5C, pore with dark red region) to account for the narrowly distributed blockade level of 26.2 ± 0.7%. Binding across the entire channel would have led to a broader spread in blockade levels (see below and Supplementary Figure S8a). We choose the dissociation rate *k*_d_ so that the simulated distribution matches the experimental data (Supplementary Section S5), achieving an excellent fit to the duration distribution in the plot (Figure 5C, dark blue dots). Not all translocation events shifted to the right given a limited association rate constant (Supplementary Figure S6e,f). While successful, the simulations did not generate the shorter cluster of experimental events with a wide distribution of blockade levels (Figure 5C, gray).

To explain the experimental data, we assumed that binding can also take place to the entire inner pore wall (Figure 5D, pore, light red region) in addition to the existing subsection of the pore (Figure 5D, pore, dark red region). The simulations hence allowed the protein to bind to either one or both of these within a single translocation event. The target was a fit to all measured events longer than 100 μs, avoiding events possibly distorted by filtering.^60^ The data were approximated well by a double-exponential distribution (Supplementary Figure S7c) with fits of *k*_d_ = 77 s^–1^ and *k*_d,2_ = 6434 s^–1^ implying that the second interaction is weaker than the first.

Visual comparison between simulated and experimental data (Figure 5D, plot) confirmed the agreement. The simulated events (Figure 5D, plot, blue) showed a broader spread of blockade level as the protein could bind to the entire pore lumen. At the wider entry of the nanopore, protein binding leads to lower and more variable current amplitudes, while binding in the narrower channel causes a more extensive blockade. As an additional point, the shorter events are more frequent as they can bind to a much larger area in the pore lumen. Third, repeated binding to various parts of the pore was also observed in exemplary current traces (Supplementary Figure S8c). A remaining difference is the less extensive blockade level in the simulated events compared to experimental data.

## Conclusions

This report has explored the scientifically and technologically relevant topic of how proteins move through nanoscale confined space. To answer important questions about transport dynamics and interactions with the pore wall, protein transport was microscopically modeled with a high-throughput computational approach to generate hundreds of millisecond-to-second protein trajectories under experimentally realistic conditions.

The multiscale simulation framework advances the field of modeling in several ways. Electroosmotic drag on the analyte is properly considered for protein transport,^17^ and the knowledge about full position-dependent diffusivity is exploited to compute ionic pore currents. Furthermore, *via* decoupling continuum calculations and the Brownian dynamics-based simulation of the trajectories, computational efficiency is achieved to easily model thousands of stochastic translocation events. Finally, analytical read-out traces are simulated almost in real time.^27−30^

The study offers fundamental insight with impact for understanding and engineering pores in sensing and research. In the case of biosensing inorganic nanopores, less than 0.05% of protein translocations lead to the desired biomolecular recognition by the cognate pore-tethered receptor. Future engineering to improve biosensor sensitivity will likely aim at increasing this percentage such as by narrowing the pore diameter and positioning the receptor at the narrowest pore part. In addition, nonspecific binding of proteins to the pore wall was examined with recently developed DNA nanopores. The analysis revealed a high extent of adsorption to the DNA pore wall based on comparing simulated and experimental data. To turn the DNA pores into valuable research and biosensing tools, the extent of nonspecific binding will have to be avoided.

We expect that additional considerable insight can be gained from constructing more complex computational biophysical models of the pores and proteins.^69−71^ This could involve pores that contain flexible parts that nanomechanically respond to biophysical stimuli or potentials. However, it is open whether such fine-grained molecular models can be reconciled with our efficient decoupling scheme, where the force field is fully sampled before the trajectories are computed. Very likely, both a much higher number of samples and a more sophisticated sampling algorithm would be needed. Another possible and easy-to-implement improvement is to include modifications in the PNPS equations that account for finite ion size.^44,68^

With minimal adaption, our computational framework can also be extended beyond nanopore sensors to model the functional behavior of porous filtration devices. Thanks to the existing embedded hydrodynamic model, the framework also applies to the filtration-relevant situations where water pressure drives molecular particles through the pore in contrast to sensing applications, where electrical potential is the driving force. In conclusion, scientific insight and simulation frameworks can empower researchers in fundamental and applied nanotechnology to gain understanding of existing systems and guide the design of pore systems.

## Methods

### Langevin Equation

The equation governing the motion of proteins is the Langevin equation:^72,73^
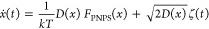
1Here, *x* is the particle position, *k* the Boltzmann constant, *T* the temperature, *D*(*x*) the (position-dependent) diffusion coefficient, *F*_PNPS_(*x*) the mean force on the protein when it rests at position *x*, and ζ(*t*) the Gaussian noise term resulting from random collision forces. Both the mean force and the diffusivity are calculated with the help of a continuum model, as explained below.

### Continuum Model

The Poisson–Nernst–Planck–Stokes equations form a coupled system of partial differential equations to describe the interaction of mobile charge carriers (ions) with the electrostatic environment and induced electro-osmotic flow of the background medium (water):
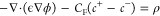
2a
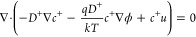
2b
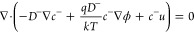
2c
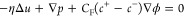
2d
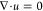
2eHere, ϵ is the material-dependent permittivity, *C*_F_ the Faraday constant, ρ the permanent charge density, *D*^±^ the ion diffusivities, *q* the elementary charge, and η the fluid viscosity. The equations are solved for the unknown electric potential ϕ, the positive and negative ion concentrations *c*^+^ and *c*^–^, the fluid velocity *u*, and the pressure *p*. Because the charge coefficients of cation (*c*^+^) and anion (*c*^–^) concentrations in these equations are +1*q* and −1*q*, respectively, the presen,t form applies to a symmetric monovalent electrolyte. Other valencies or a different number of ion species could be accommodated similarly.

The three-dimensional computational domain includes the pore and membrane which are surrounded by an electrolyte reservoir; see Figures 2a, 3a, and 4a. A detailed description of the computational model including boundary conditions can be found in our previous work;^38^ that work also introduces the iterative solution method for the PNPS system, which we deploy here.

As opposed to ions and water molecules, which are only implicitly modeled as a continuum, the protein is explicit in our model and occupies a part of the computational domain. From one simulation with the protein at a fixed position *x*, we obtain the ion current through the pore as well as the PNPS force acting on the protein at this position. The ion current is given by
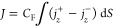
where *j*_*z*_^±^ is the *z*-component of the flux density:
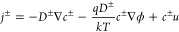
The integral is taken over any horizontal cross-section of the pore. The PNPS force is given by *F*_PNPS_ = *F*_el_ + *F*_drag_, where
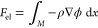
3a
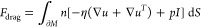
3b*M* is the volume occupied by the protein, ρ is the charge density, and *n* is the surface normal. Current and force are computed for several hundred positions of the protein and stored in a look-up table, which is then used, by interpolation, to obtain their values at arbitrary positions of the domain. A streamline plot of the resulting global force field is shown in Supplementary Figure S4.

### Position-Dependent Diffusivity

The ion diffusivities *D*^±^ in eqs 2 determine the strength of ionic currents as predicted by our model, while the protein diffusivity enters eq 1 and determines the translocation speed. Diffusivity at any position of the domain is given by a 3 × 3 tensor corresponding to the three spatial dimensions; it thus depends on the position as well as the direction of motion. For example, close to a wall, diffusivity in general is reduced considerably, and motion perpendicular to the wall is affected more strongly than motion in the other two directions.^54^

A full hydrodynamic model for the computation of the diffusion tensor makes use of the numerical solution of the Stokes equations. It is based on a generalization of Stokes’ law for the drag force on a spherical particle suspended in water:
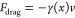
4Here, *v* is the particle velocity and γ(*x*) is the position-dependent *friction (or resistance) tensor*, which relates velocity and drag force. To compute γ(*x*), we can place the target molecule at position *x* and solve the Stokes equation with *v* as the no-slip boundary condition at the molecule boundary, *i*.*e*.,
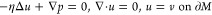
5where symbols are defined as in eqs 2. The drag force is calculated from the resulting velocity field *u* as in eq 3b. Knowing about the linear relationship eq 4, which holds in general, we can obtain the full 3 × 3 friction tensor from three evaluations of *F*_drag_, *e*.*g*., for velocities *v* = (1, 0, 0), (0, 1, 0), and (0, 0, 1). Finally, the diffusion tensor is computed from the friction tensor *via* the Einstein relation^74^
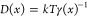
6

In principle, *D*(*x*) can in this way be obtained for arbitrary protein and ion positions, and many evaluations could be interpolated to obtain global diffusivity fields. We call this the low Reynolds-number hydrodynamcs method. In practice, this is computationally infeasible, especially for ion diffusivities where the geometry and hence the entire diffusivity field depend on the particular protein position. For our simulations, we have therefore relied in part on analytical approximations available for simplified geometries, as detailed in Supplementary Section S2.

### BD Algorithm

Based on the global diffusivity and force fields obtained with our continuum model, we compute protein trajectories with the Langevin eq 1. In its discretized form,^75^ this becomes the updated equation
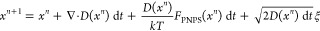
7where *x*^*n*^ and *x*^*n*+1^ are successive protein positions, d*t* is the size of the time step, and ξ is a vector of three standard normally distributed random numbers. Since *D* is a (positive semidefinite) matrix, a valid square root  is any matrix *C* satisfying *D* = *CC*^*T*^, for instance obtained *via* Cholesky factorization. The divergence ∇·*D* is calculated numerically from the global diffusivity field. When a position update leads to the protein penetrating a wall, we shorten the step so that the protein comes to stop right before the wall, which models hard sphere reflections at the microscopic level.

### Protein–Pore Binding

To implement protein adsorption on top of the BD algorithm, we first define a *binding site*, which can be part of the pore wall or a spherical receptor close to the wall. Then, we have to choose a binding radius *r*_b_. The *binding zone* is the set of protein locations where the distance from protein center to binding site is smaller than *r*_b_. Next, we need to know the adsorption rate inside the binding zone, which is denoted by *R*_a_ and has the units of 1/s. It is related to the association rate constant *k*_a_, which would be obtained from kinetic bulk measurements of the same interaction, by *R*_a_ = *k*_a_*c*_b_, where *c*_b_ is the concentration of receptors inside the binding zone. In the case of an isolated spherical receptor, the binding zone is a spherical shell with volume , where *r*_prot_ and *r*_rec_ are the radii of the protein and receptor. The receptor concentration is just the inverse of this volume, converted to moles per liter, *i.e.*, *c*_b_ = (10^3^*N*_A_*V*_b_)^−1^, where *N*_A_ is Avogrado’s constant. Hence, we find
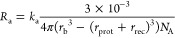
8If the binding site is a part of the pore wall and nothing is known about the interaction, we will just set *r*_rec_ = 0 and use eq 8 as the defining relationship between *R*_a_ and *k*_a_.

The likelihood of binding is determined by *R*_a_ and the time spent in the binding zone, which we call *attempt time*. In every BD time step, we check whether the protein is in the binding zone; if so, we count that as an attempt time equal to the length of the time step d*t*. The number of adsorptions during the time step is then drawn from a Poisson distribution with mean *R*_a_ d*t*. The Poisson distribution follows from the assumption of a first-order reaction between proteins and receptor, while the protein is in the binding zone.

We note that our approach is a refinement of the one taken in the pioneering work on nanopore adsorption by Sexton *etal*.,^45^ where, when formulated in our terms, the entire pore was treated as the binding zone. In our work, in contrast, the binding zone is only a small shell around the receptor, which the protein only enters when the circumstances of pore geometry and applied forces allow it.

For each adsorption event, the binding duration τ (time until desorption) is a stochastic variable depending on the (bulk) dissociation rate *k*_d_. In the simplest case where we assume no dependence on applied force, τ is drawn from an exponential distribution with mean τ̅ = τ_0_ := *k*_d_^–1^. This approach was taken for the DNA origami nanopore. To implement force dependence, an additional parameter is needed: the “bond rupture length” δ. Then, binding duration is drawn from an exponential distribution with mean
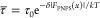
9where the PNPS force on the protein at its current position appears in the exponent. In practice, the parameter δ has to be determined inversely from current event data measured at different applied voltages. This is done for the receptor-modified solid-state nanopore in Supplementary Figure S3c.

### Summary: Calculation of Protein Trajectories

With all submethods in place, let us summarize our end-to-end algorithm for computing protein trajectories with binding.

- *Step 1: Preparation.* Build a computational model of the geometry; create a grid of several hundreds of protein positions *x*_*i*_ that are physically possible (the protein does not overlap with the channel walls or membrane) and that cover the domain on which trajectories are to be investigated.
- *Step 2: Continuum simulations.* For each grid position *x*_*i*_,
  (a) create a finite element model that includes the spherical protein centered at *x*_*i*_ and that contains boundary conditions that account for applied voltage and partial charges;
  (b) use the PNPS finite element solver to compute several physically relevant scalar and vector fields: electric potential ϕ, cation and anion concentrations *c*^+^ and *c*^–^, fluid velocity *u* and pressure *p*;
  (c) compute numerical integrals that yield the force vector *F*_PNPS_(*x*_*i*_), which acts on the protein, as well as the current through the pore *J*(*x*_*i*_). Store these values in a table alongside the position *x*_*i*_.
- *Step 3: Preparation, part 2.* Postprocess the grid values *F*_PNPS_(*x*_*i*_) and *J*(*x*_*i*_) into a data structure that enables fast interpolation to calculate *F*_PNPS_(*x*) and *J*(*x*) at arbitrary positions *x* of the computational domain. Furthermore, create a protein diffusivity field (Supplementary Section S2), which also can be evaluated at arbitrary positions to yield *D*(*x*).
- *Step 4: Brownian dynamics trajectories.* Repeat the following steps several hundreds to thousands of times (depending on the number of trajectories needed):
  (a) Compute a protein trajectory by using the BD algorithm. Three main factors determine the shape of this trajectory: the precomputed force and diffusivity fields, the confining geometric elements of the channel, and the randomly drawn collision forces (which ensure variation between trajectories). For every step where the protein is within the binding zone of a receptor, store the time *t*, position *x*(*t*), and force *F*_PNPS_(*x*)—which are also used by the BD algorithm—for later use in the binding algorithm. (In the case of multiple different kinds of binding, multiple such lists are created.)
  (b) Compute the current trace by evaluating *J*(*x*) at every trajectory position. If needed for visualization, store the entire current trace; otherwise, store aggregated results: the total duration and average current amplitude without binding, and the current amplitude at the potential binding positions of step a).
- *Step 5: Protein–pore binding.* For each trajectory from step 4, do the following:
  (a) For each recorded time step where the protein was in a binding zone, randomly draw the number of bindings in that step from a Poisson distribution with mean *R*_a_ d*t*. For each of these bindings, compute the mean adsorption time as in eq 9 (possibly using the force *F*_PNPS_(*x*) recorded in step 4) and randomly draw the binding duration from an exponential distribution around this mean.
  (b) Compute the total event duration τ_off_ by adding up the binding durations (if any) obtained in the last step and the total time without binding. Compute the current amplitude *A*/*I*_0_ as a time-weighted average over the amplitudes at binding locations and the average amplitude without binding.

An important thing to note is that stochastic binding events (step 5) are computed in a separate step, after all trajectories were computed in step 4. This is possible because a trajectory’s spatial shape is not influenced by a binding event. It also means that, instead of using each trajectory exactly once, as in step 5, we can use the following variant:

- *Step 5′: Protein–pore binding (alternative variant).* Repeatedly choose a random trajectory out of all trajectories computed in step 4. Perform steps 5a and 5b to compute event duration and amplitude.

In this version, every trajectory is potentially used many times, and each time produces a different event duration and amplitude (since binding durations are stochastic). Step 5′ is a possible way to create more current events, since the drawing of binding events is much cheaper than computation of a trajectory. However, it is important in this case that the number of trajectories created in step 4 is large enough, to avoid sampling from a distorted distribution of trajectories. We used this variant for the solid-state pore, where binding was very rare. First, 100 000 trajectories were computed in step 4, and then several tens of millions events where drawn in step 5′. This large number was necessary to observe about 500 events with binding, the same number as in the experiments.

### Model Parameters

The following is a list of physical parameters and constants used throughout all our simulations:

- Boltzmann constant *k* = 1.3806 × 10^–23^ J/K, temperature *T* = 293 K
- Viscosity of water η = 10^–3^ Pa s
- Vacuum permittivity ϵ_0_ = 8.854 × 10^–12^ C/(V m)
- Relative permittivies: water 80.2, protein 2, DNA 12, lipid membrane 2, silicon nitride 7, gold 6.9, SAM layer^35^ 2.7
- Avogrado constant *N*_A_ = 6.022 × 10^23^ mol^–1^, elementary charge *q* = 1.602 × 10^–19^ C, Faraday constant *C*_F_ = *qN*_A_
- Hydrodynamic radius of both K and Cl ions, used in hydrodynamic calculations: *r* = 0.11 nm. The value is chosen so that the bulk diffusivity derived from Stokes’ law, , matches experimental values.

Next, we list parameters specific to the simulations for each particular pore.

α-Hemolysin:

- Pore geometry and charge distribution were constructed with the help of protein modeling software; see Supplementary Section S1. The channel is 10 nm long with radii ranging from 0.5 to 2.8 nm.
- The lipid membrane is centered at a height of −7.6 nm relative to the upper channel entry, is 2.2 nm thick, and carries no surface charge.
- Dimensions of the computational domain (cylindrical water reservoir): height 22 nm, radius 10 nm
- Bulk concentration of ions: 1 M

Solid-state pore:

- Membrane thickness: silicon nitride 50 nm, gold film (vertical direction) 40 nm, gold film (radial direction) 10 nm, thickness of the SAM layer 3 nm
- Channel is conical with an aperture of 40°; pore diameter at the tip (smallest diameter) is 20 nm in Figure 3b and 24 nm in Figure 3c (same as in corresponding experiments)
- Dimensions of computational domain (cylindrical water reservoir): height 240 nm, radius 120 nm
- Surface charge densities: silicon nitride −0.022 C/m^2^, SAM layer −0.078 C/m^2^, gold 0
- Bulk concentration of ions: 1 M
- Applied voltage: 200 mV in Figure 3b, varied in Figure 3c
- Protein A/G/L is a sphere of radius 3 nm and charge of −50*q*
- Receptor location is fixed at 95*%* of channel height, at 2.75 nm distance from the wall.
- Proteins start their trajectory at random positions on the disc described by the larger channel entrance.
- Size of BD time step: *dt* = 1 ns
- Binding radius (relative to centers of protein and receptor): 5.75 nm
- Binding constants from Lata *etal*.:^59^*k*_a_ = 1.5 × 10^5^/(M s), *k*_d_ = 25 × 10^–3^/s
- Binding constants estimated from Wei *etal*.:^35^*k*_a_ = 5.2 × 10^6^/(Ms), *k*_d_ = 4.5 × 10^–3^/s; estimated effective bond rupture length: δ = 0.55 nm (see Supplementary Section S3 for the estimation methodology)

DNA pore:

- The geometry is constructed by modeling each DNA strand as a stiff rod with square, 2 × 2 nm^2^ cross-section. This results in a box-like channel with a diameter of 6 nm, wall thickness of 6 nm, and channel length of 46 nm.
- Lipid membrane is attached at the channel bottom, 2.2 nm thick and uncharged.
- Dimensions of computational domain (box-shaped water reservoir): 20 × 20 × 70 nm^3^
- Surface charge density of DNA (if not otherwise stated): 0.74 *q*/nm^2^
- Bulk concentration of ions: 1 M
- Applied voltage: 80 mV, except in Figure 4c, where it is 100 mV
- Protein trypsin is a sphere of radius 2.078 nm and charge +5*q*.
- Proteins start their trajectory centered at the upper channel entry.
- Size of the BD time step: d*t* = 0.2 ns
- Binding radius for both types of binding (relative to center of protein and wall): 0.2 nm
- Binding constants are fit to experiments, see Supplementary Section S5.

## Supporting Information Available

Details on models, simulations, and parameter estimation (PDF)
