# Supplementary material for: Protein Transport through Nanopores Illuminated by Long-Time-Scale Simulations
Source: ACS Nano. 2021 Jun 7;15(6):9900–12. doi: 10.1021/acsnano.1c01078 (PMC8291773; doi:10.1021/acsnano.1c01078)
Supplement: Supplementary file 1 — nn1c01078_si_001.pdf [file nn1c01078_si_001.pdf]

Supplementary Information

Protein Transport through Nanopores Illuminated by Long-Timescale Simulations

Gregor Mitscha-Baude, Benjamin Stadlbauer,
Stefan Howorka*, Clemens Heitzinger*

E-mail: s.howorka@ucl.ac.uk, clemens.heitzinger@tuwien.ac.at

Contents

S1 Axisymmetric model of α-hemolysin 2

S2 Calculation of position-dependent diffusivity 3

S3 Protein-receptor binding in solid-state pore 5

S4 Model of DNA origami pore 8

S5 Non-specific binding to DNA nanopore 9

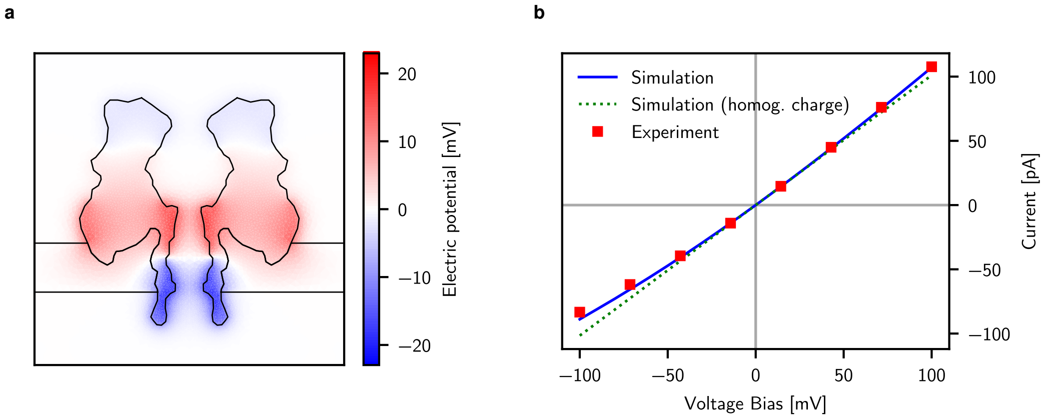

Fig. S1 | Axisymmetric model of α-hemolysin. a, Electrostatic equilibrium potential illustrating the location of partial charges. **b**, IV curves in 1 M KCl; experimental values are compared with two different models for surface charge; diffusivity is constant in the channel and fitted to the measurements.

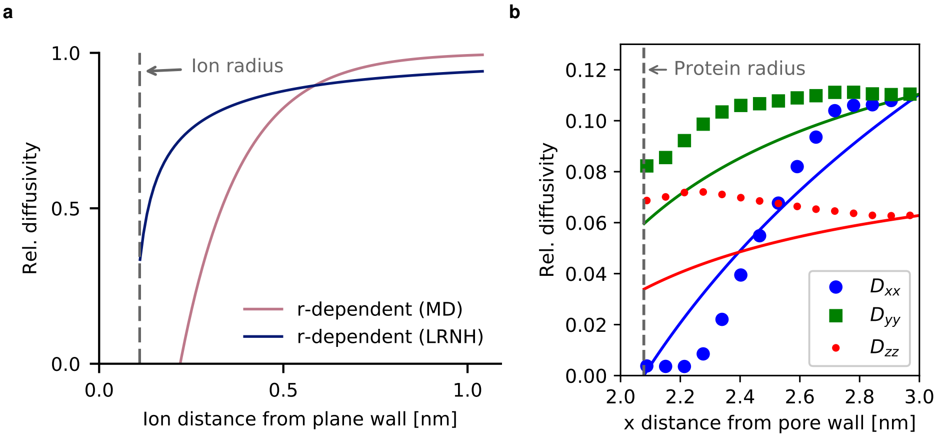

Fig. S2 | Position-dependent diffusivities. a, r-dependent tangential diffusivity near a plane wall, hydrodynamic (LRNH) approximation D_t *vs.* analytical expression $1 - e^{-a(r-r_0)}$ with parameters a, r_0 fit to MD simulations.[2] The MD data are collected from various simulation of ions and water molecules near DNA and protein,[3] where the diffusing molecule was confined from one side. **b**, DNA nanopore, same as Figure 4b (main text) but for diffusivity of protein trypsin.

S1 Axisymmetric model of α-hemolysin

Figure S1a shows our axisymmetric model of the α-hemolysin pore together with the electrical potential at zero voltage. Partial charges are incorporated by dividing the pore protein into 4 vertically aligned pieces and equi-distributing the charge contained in each piece over the respective part of the surface. This way we can reproduce the current rectification of the channel, as shown in Fig. S1b. The experimental IV data for a 1 M KCl solution were taken from ref.[1] We also compare with a simpler model where the surface charge is constant over the *whole* protein surface (not divided into 4 pieces). This homogeneous charge model yields an ohmic current-voltage relationship and fails to capture rectification. To make this result clearly visible in Fig. S1b, we used a constant diffusion coefficient inside the pore that fits the observed current ($D = 0.3D_0$ where D_0 is the bulk value)—as opposed to the main text (Fig. 2b), where we use parameter-free models for the diffusivity.

S2 Calculation of position-dependent diffusivity

The most direct method to compute the diffusivity tensor at any position is by numerically solving the Stokes equation, with the particle of interest included explicitly in the geometry, as explained in the Methods. For our simulations, we combined this direct method with different variants of approximations.

z-dependent model In the first variant, we compute the diffusivity tensor numerically, but only at positions along the central pore axis. This yields a one-dimensional diffusivity profile as depicted in Fig. 2c (main text) for α-hemolysin. The dependence of diffusivity on other coordinate directions is ignored. Outside the channel and small regions close to its entrances, bulk diffusivity is used.

r-dependent model This variant relies on the analytical treatment of a simple geometric situation, that of a solid sphere next to an infinite plane wall. For a sphere of radius a whose center of mass is located at a distance r from the plane, Happel and Brenner[4] give the following expressions in terms of $x := a/r$ and $\alpha := \cosh^{-1}(r/a)$:

$$
\begin{aligned}
D_t &= 1 - \frac{9}{16}x + \frac{1}{8}x^3 - \frac{45}{256}x^4 - \frac{1}{16}x^5, \\
D_n^{-1} &= \frac{4}{3}\sinh\alpha \sum_{n=1}^{\infty} \frac{n(n+1)}{2n(2n+3)} \left[\frac{2\sinh(2n+1)\alpha + (2n+1)\sinh 2\alpha}{4\sinh^2(n+\frac{1}{2})\alpha - (2n-1)^2\sinh^2\alpha} - 1 \right].
\end{aligned}
$$

Here, D_t and D_n are the relative diffusivities for motion parallel and perpendicular to the plane, respectively. The full 3×3 diffusivity tensor for arbitrary orientations of the plane and particle is given by

$$D = D_0 \left[D_n \mathbf{R}\mathbf{R}^T + D_t (I - \mathbf{R}\mathbf{R}^T) \right],$$

where $\mathbf{R}$ is the normal vector pointing from the plane to the particle and $D_0 = \frac{kT}{6\pi\eta a}$ is the bulk diffusivity. For consistency of the theory with measured bulk diffusion constants of ions, we use their hydrodynamic radius for a in all these expressions.

The framework above can approximately be applied to a complicated geometry by considering only the distance and normal vector to the *nearest wall* for any particle position, and computing diffusivity as if that wall were replaced by its tangent plane (and no other walls present). To implement this, we calculate the wall-distance r by numerically solving the Eikonal equation

$$|\nabla r| = 1, \qquad r = 0 \text{ at the wall,}$$

inside the fluid domain. The normal vector pointing from the nearest point at the wall to any particle position is simply $\mathbf{R} = \nabla r$. By computing r and $\mathbf{R}$ over the whole domain and then using the formulas for D above, we obtain a global, r-dependent diffusivity field for a particle with given radius a. This model is also applied to ions if the “nearest wall” is the target protein.

r-/z-dependent model The r-dependent model is practical as it yields a reasonable value for the diffusivity tensor at arbitrary points of the domain, but suffers from the fact that in a narrow channel, diffusivity can be quite a bit lower than if there was a wall only on one side of the particle. Therefore, we correct the r-dependent model so that it equals the true diffusivity at the pore center, *i.e.*, combine it with the z-dependent model. This is achieved by setting

$$
D(x,y,z) := \begin{cases} D_r(x,y,z) D_r(0,0,z)^{-1} D_z(z), & \text{inside channel region,} \\ D_r(x,y,z), & \text{elsewhere,} \end{cases}
$$

where D_r and D_z are the diffusivity tensors computed by the r- and z-dependent models, respectively.

Modified model for proteins in DNA pore The nearest-wall approximation works very well in the case of ions, which are small compared to the channel (see Fig. 4b, main text), and for the same reason also for protein A/G/L in the large solid-state nanopore. But it turns out to fail when applied to protein trypsin in the DNA nanopore, where the protein diameter is about two thirds of the channel width. In this case, non-nearest-wall effects completely change the diffusion profile, especially for motion parallel to the channel; see Figure S2b. Similar results were obtained experimentally.[5] For this case, we use the r-/z-dependent model from above only outside and in the wider entry of the channel. In the narrow channel part we interpolate the profile obtained from simulations (symbols in Fig. S2b) and use that instead of the analytical expressions for D_n and D_t to define the diffusivity tensor, assuming the profile is constant over the narrow channel part.

S3 Protein-receptor binding in solid-state pore

For the solid state-pore, the geometry is modeled after the estimates by Wei *et al.*;[6] they describe a conical pore of 40° aperture situated in a 50 nm thick silicon nitride membrane, coated by a 40 nm gold film and a SAM layer of estimated thickness 3 nm. The pore diameter (at the pore tip, without SAM layer) is $d_p = 26$ nm for the simulations in Fig. 3b (main text) and $d_p = 30$ nm in Fig. S3b, corresponding to the reported experimental diameters in Fig. 2b and 3a of ref.,[6] respectively. Protein A/G/L was modeled as a sphere of radius 3 nm and charge $-50q$, while the bis-NTA receptor is not explicitly modeled, only implicitly as a location in the pore where the protein can bind. The binding radius (distance between the protein center and receptor within which binding is possible) was set to 5.75 nm. In Fig. S3a, we show a histogram of the total attempt time, *i.e.* the time the protein spends within the binding radius of the receptor during one simulation; it roughly resembles an exponential distribution. Trajectories with zero attempt time, which comprise 79% of all simulations, were excluded.

Voltage-dependent dissociation To simulate the variation of k_{off} in dependence of applied voltage, we adopt the binding model with force-dependent dissociation rate; see Methods, Eq. (9). The parameter δ (the "bond rupture length") of this model has yet to be determined. For each voltage, we generated a plot of the cumulative τ_{off} distribution as depicted in Fig. S3b. The mean binding duration $\overline{\tau_{\text{off}}}$ was determined by fitting a cumulative exponential distribution $1 - e^{-\tau_{\text{off}}/\overline{\tau_{\text{off}}}}$ to these values (nonlinear least-squares fit as described in Section S5 below). By setting $k_{\text{off}} := 1/\overline{\tau_{\text{off}}}$, we generate an estimate of k_{off} for every applied voltage, resulting in a plot like Fig. 3c (main text). Then, an empirical relationship $k_{\text{off}} = k_{\text{off}}^{V=0} e^{\alpha V}$, where V is the applied voltage, can be found by fitting the straight lines in this plot. The constant α—which has dimension $1/V$ and describes the sensitivity of k_{off} to applied voltage—can be compared to the same constant obtained from measurements in ref.[6] In our simulation, α depends on the chosen value of δ (in a roughly linear way, as can be seen in Fig. S3c). However, α is also influenced by the location of the receptor: the closer the receptor is to the pore tip, the higher the sensitivity of k_{off} to applied voltage, because the electric field is strongest at the tip. We follow the argumentation in ref.[6] that the receptor is suspected to reside close to the tip, but allow some uncertainty by performing calculations for a receptor at 91%, 93%, 95% and 97% of the pore height, respectively. Similarly, Wei *et al.* provide data for five different pores which show a slight variation in α. Both sets of data are compared in Fig. S3c, where the parameter δ is varied in the simulations and averages are plotted along each set of data points. We see that both the average and width of the distributions agree at $\delta = 0.55$ nm, so this value emerges as our estimate (and is in fact used in all other plots). While the order of magnitude of δ is consistent with its interpretation as a bond rupture length, we stress that this interpretation relies on an oversimplified picture of the interaction.

The choice of δ (together with the receptor position) only fixes the slope in the k_{off}-voltage plot (Fig. 3c, main text). The absolute height, indicated by the extrapolated value at zero voltage, $k_{\text{off}}^{V=0}$, is essentially the dissociation rate k_d, which we took from Lata *et al.*[7] Alternatively, we can also fit k_d directly to the experiments, as we did with δ. This provides validation that our binding model can, in principle, reproduce the distribution of event durations. To this end, in Fig. S3d, we vary k_d in our simulations to find a value that leads to agreement in $k_{\text{off}}^{V=0}$ with Wei *et al.*[6] Effectively, we see that $k_{\text{off}}^{V=0} = k_d$. The rate $k_d = 4.5 \cdot 10^{-3}$/s obtained in this way leads to very close fits of the experimental data; see the simulations labeled k_d *from Wei* in Figures 3b and 3c (main text).

Association and arrival rate Next, we turn to the association rate constant k_a. Lata *et al.* report $k_a = 1.5 \cdot 10^5\,\text{M}^{-1}\text{s}^{-1}$ for solution measurements involving different molecules attached

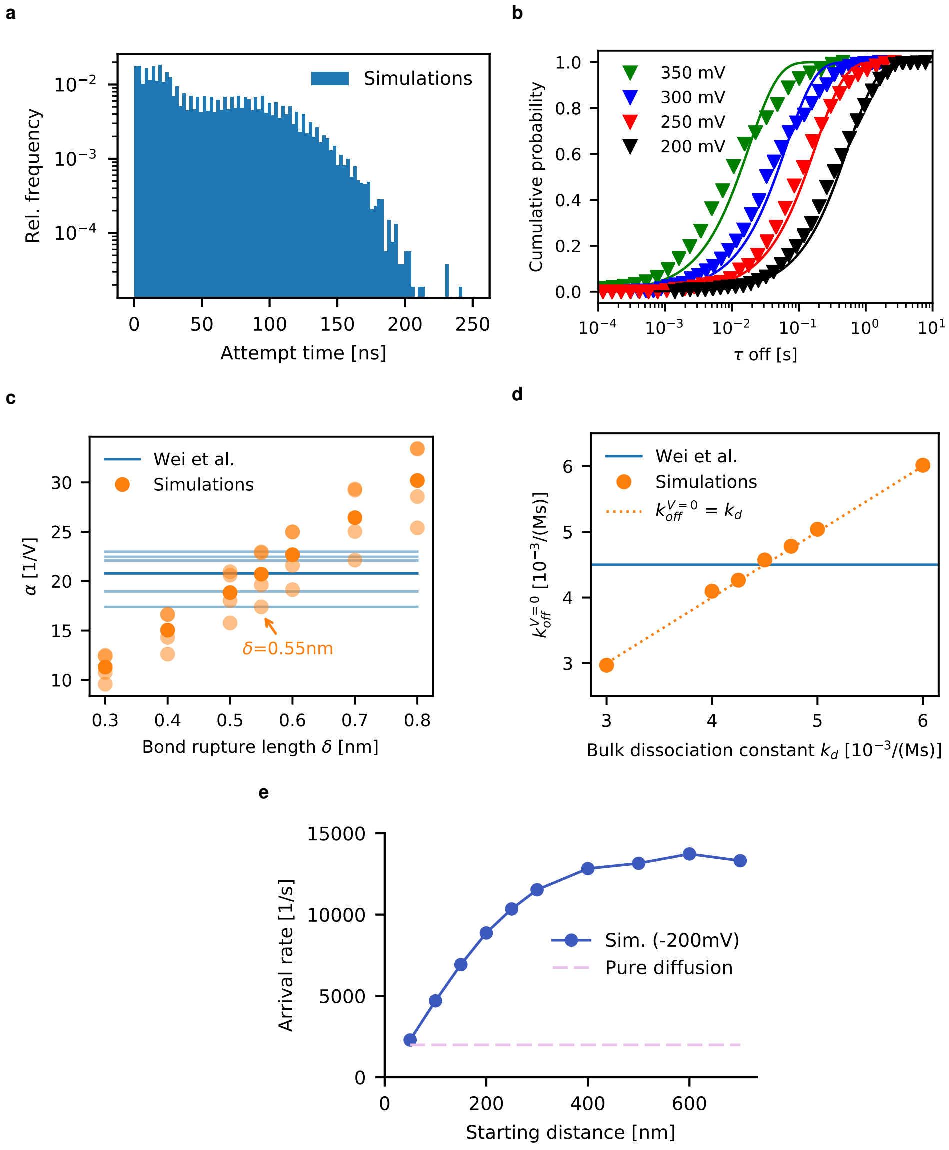

Fig. S3 | Parameter estimation of protein-receptor binding. **a**, Histogram of simulated attempt times (*i.e.*, the times a translocating protein spent in the binding zone around the receptor). Applied voltage is 200 mV. **b**, Cumulative histograms of simulated $\tau_{\rm off}$ (total dwell time), fitted with exponential distributions. The exponential fits are used to obtain characteristic dwell times $\overline{\tau_{\rm off}}$. **c**, Estimation of parameter δ by fitting to the slope α of the $k_{\rm off}$-voltage relationship. Light blue lines are measurements obtained from several different pores (Fig. 3b in ref.[6]), light orange dots are simulations for three different receptor positions. Darker line/dots are averages. **d**, Estimation of parameter k_d by fitting to $k_{\rm off}^{V=0}$. **e**, Estimation of arrival rate k_{arr} by combining Smoluchowski's equation with simulations of proteins migrating to the pore entrance.

to the reaction sites, which cannot be expected to transfer well to the present setup. We can relate k_a to measurements of $\tau_{\rm on}$, which is the time between current blockades. The inverse $1/\overline{\tau_{\rm on}}$ is the rate at which new current blockades are observed when the pore is in its unoccupied state. Wei *et al.* find that this rate increases linearly with protein concentration c, in symbols $1/\overline{\tau_{\rm on}} = c \cdot k_{\rm on}$, and they report an average value of $k_{\rm on} = 20.9 \cdot 10^6\,{\rm M}^{-1}{\rm s}^{-1}$ from 5 measurements at different concentrations.

It is important to stress that in our definition of the binding model, $k_{\rm on}$ does not equal k_a in general. $k_{\rm on}$ is the apparent association rate constant when considering the protein-nanopore interaction as a whole, but it likely depends on features which are unrelated to

the receptor, such as the voltage bias. By comparison, in our model, k_a is designed to be the microscopic association rate constant intrinsic to the analyte and receptor. We would expect $k_a = k_{\text{on}}$ only if unhindered and unbiased diffusion would govern protein migration to the receptor.

To find the relationship between k_a and $\overline{\tau_{\text{on}}}$, we first note that our simulation yields an average number of bindings per event, given by $k_a \frac{t_b}{V_b}$ where t_b is the mean time the protein spent in the binding zone, and V_b is the binding zone volume (in units of M^{-1}). Second, let k_{arr} denote the the rate of protein arrival at the pore, *i.e.* the total rate of events per second (this will be calculated below). Then we have (bindings per second) = (bindings per event) $\times$ (events per second),

$$1/\overline{\tau_{\text{on}}} = k_a \frac{t_b}{V_b} k_{arr}$$

In the simulation at $-200\,\text{mV}$, we find $\frac{t_b}{V_b} = 5.32 \cdot 10^{-11}\text{Ms}$, while measurements give $\overline{\tau_{\text{on}}} = 0.265\,\text{s}$ at the same voltage and a protein concentration of $180\,\text{nM}$. Dividing the last equation by k_{arr}, we arrive at two ways to compute the number of bindings per event, which have to be equal to be consistent with measurements at $-200\,\text{mV}$:

$$\text{bindings per event} = \frac{3.76}{k_{arr}} = 5.32 \cdot 10^{-11} k_a \tag{1}$$

The value $k_a = 1.5 \cdot 10^5\,\text{M}^{-1}\text{s}^{-1}$ from Lata *et al.* yields a binding percentage of 0.0008% which implies an arrival rate $k_{arr} = 470000\,/\text{s}$. To compute a more realistic k_a, we take the reverse approach and estimate k_{arr} directly.

A simple analytical formula for the arrival rate at a half-sphere covering the pore entrance is the Smoluchowski rate equation:[8–10]

$$k_{arr} \approx 2\pi DcR$$

where D and c are the protein's diffusivity and concentration (in m^{-3}) and R is the pore radius at the entrance. Applying the formula to our pore with a radius at the bottom of $41\,\text{nm}$ yields $k_{arr} = 1990\,/\text{s}$.

However, the Smoluchowski equation is only valid for unbiased diffusion. Since in the measurement data we have considerable electrophoretic bias towards the pore, the equation underestimates the arrival rate. To correct for bias, we made use of our simulation methods and computed a more realistic k_{arr} in two steps: First, compute the arrival rate with the Smoluchowski equation at a distance R much larger than the pore radius, where the assumption of unbiased diffusion is valid. Second, simulate PNPS/BD trajectories with the protein starting at the larger distance (randomly distributed on a half sphere), to compute the probability for a protein to transition to the pore entrance. The arrival rate at large distance multiplied with the transitioning probability gives the arrival rate at the entrance.

This was done for successively larger distances, see Figure S3e (blue circles). We can see that the arrival rate is increasing with increasing distance, reflecting the bias near the pore, until at $400-600\,\text{nm}$ the curve flattens. The largest value (a lower bound on the actual arrival rate) is $k_{arr} = 13700\,/\text{s}$ which is $7\times$ the rate we got from naively applying Smoluchowski's equation with R equal to the pore radius (Figure S3e, dashed pink line).

Using equation (1) we arrive at a binding fraction of 0.027% and estimate of $k_a = 5.2 \cdot 10^6\,\text{M}^{-1}\text{s}^{-1}$, lower than the directly computed $k_{\text{on}} = 20.9 \cdot 10^6\,\text{M}^{-1}\text{s}^{-1}$ but still considerably larger than the $1.5 \cdot 10^5\,\text{M}^{-1}\text{s}^{-1}$ from solution measurements.

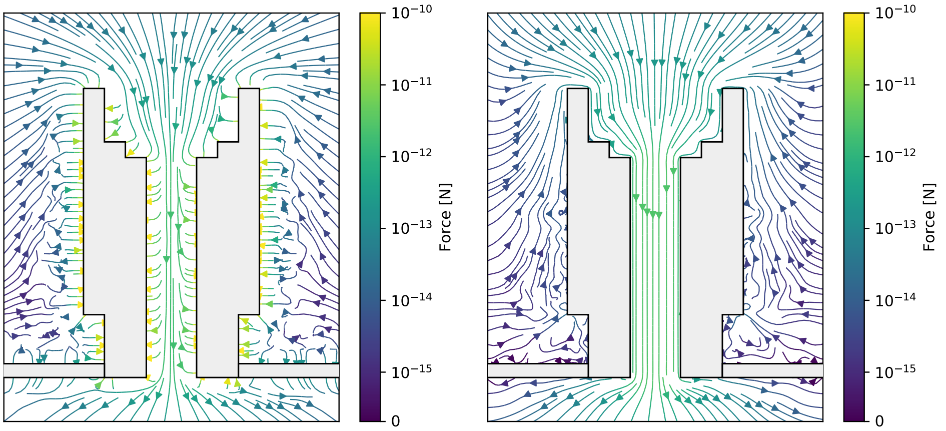

Fig. S4 | Streamline plots of force fields acting on protein trypsin in the DNA nanopore. Applied voltage is -100 mV. Left: Electrophoretic force. Right: Electroosmotic drag force.

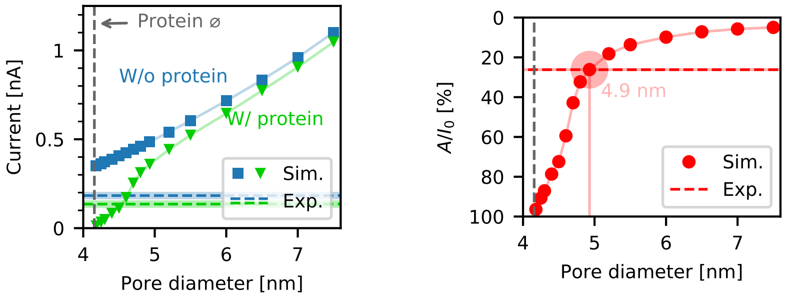

Fig. S5 | Dependence of simulated ion current on pore diameter. Same as Fig. 4d,e in the main text, but for a cylindrical model of the nanopore. In this case, the current blockade can be up to 100% if the radius of pore and protein are equal.

S4 Model of DNA origami pore

The DNA origami pore is modeled after the descriptions in ref.[11] If we assume that each DNA strand can be effectively modeled as a stiff rod with square, $2 \times 2\,\mathrm{nm}^2$ crosssection, the pore has a box-like shape with a channel width of 6 nm and a wall thickness of 6 nm. For simulations with a different channel width, the wall thickness is left the same. At the opening, the pore is wider to facilitate entry of analyte molecules. We also consider an axisymmetric version of the geometry where the pore has the same crosssection in the x-z plane but a circular cross-section in the x-y plane, *i.e.* the core channel is a cylinder. The electrolyte solution is 1 M KCl. The target protein trypsin is modeled as a sphere of radius 2.078 nm and a total charge of $+5q$ (the pH value in experiments is 8.0). The DNA surface, if not stated otherwise, is equipped with a homogeneous negative charge density of $-0.74q/\mathrm{nm}^2$, a value obtained by averaging the $-2q$ charge from two phosphate residues per 0.34 nm over the surface of each individual DNA rod. In Fig. S4, we visualize the two components of the force field (electrophoretic and electroosmotic), which were computed by our continuum method, at an applied voltage of -100 mV. For this pore and protein, the net driving force contributed by both components is in the same direction and of comparable strength: When integrated from the top to the bottom of the channel, the electrophoretic force accounts for a potential energy drop of $18kT$, while the drag force contributes $26kT$.

S5 Non-specific binding to DNA nanopore

Parameter estimation Statistical model fitting, as used for Fig. S3b and throughout this section, is based on the following general algorithm: Given an empirical data set $D = \{\tau_0, \tau_1, \ldots\}$ (*e.g.*, dwell time measurements), we first evaluate the empirical cumulative distribution function (ECDF) at a set of grid points which are logarithmically spaced over the same range as the data. Mathematically, we create $N+1$ grid points $t_j = \tau_{\min}^{1-j/N} \tau_{\max}^{j/N}$ where $\tau_{\min}$ and $\tau_{\max}$ are the minimum and maximum of the data set; and evaluate $x_j = \mathrm{ECDF}(t_j)$, where

$$\mathrm{ECDF}(t) := \frac{\#\{\tau \in D : \tau \leq t\}}{\#D}.$$

Then, suppose we have a model $F(t;\theta)$ for the CDF which depends on a vector of parameters θ. We fit the model to the empirical CDF using (non-linear) least squares, which means we search for a value of θ which minimizes the cost function

$$L(\theta) = \sum_{j=0,\ldots,N} |F(t_j;\theta) - x_j|^2. \tag{2}$$

Parameter search is performed with a simple evolutionary algorithm. Given an initial parameter vector θ and an initial standard deviation σ, we do the following:

1. Generate a new population by drawing from a lognormal distribution $\theta e^{\sigma X}$, where X is (multivariate) standard normal.

2. Replace θ by the member of the population which minimizes $L(\theta)$.

3. Reduce σ by a constant factor (*e.g.*, 0.8) and go back to 1.

The number of iterations is chosen such that σ will become sufficiently small, *e.g.* ensuring $\sigma \leq 10^{-3}$ in the last iteration. A typical population size is 100. Advantages of this algorithm include that it is easily implemented, for an arbitrary number of parameters; and that it can be applied to any model whose CDF can be evaluated, including models which are not differentiable in θ and where the evaluation of the CDF is itself stochastic; see examples below.

Long binding We fit our binding model to the current events, where events with $\tau_{\mathrm{off}} \leq 2\,\mathrm{ms}$ are cut off; see Fig. S7a. First, we want to establish that the distribution is close to an exponential one. Naively, we could apply the fitting method outlined above to the CDF

$$F(t;\overline{\tau_{\mathrm{off}}}) = 1 - e^{-t/\overline{\tau_{\mathrm{off}}}}.$$

The only parameter to be fitted is the mean dwell time $\overline{\tau_{\mathrm{off}}}$. However, because the empirical data is cut off, we likely obtain a better fit if we assume that the exponential distribution is cut off at 2 ms as well. In general, the CDF of a *truncated distribution*, which is cut off from below at t_0, has the form

$$F_{\mathrm{trunc}}(t) = \begin{cases} \frac{F(t)-F(t_0)}{1-F(t_0)}, & \text{if } t > t_0, \\ 0, & \text{else}, \end{cases} \tag{3}$$

where $F(t)$ is the non-truncated CDF (and we have suppressed the dependence on parameters). In the case of our exponential distribution, this works out as

$$F_{\mathrm{trunc}}(t;\overline{\tau_{\mathrm{off}}}) = \frac{(1-e^{-t/\overline{\tau_{\mathrm{off}}}}) - (1-e^{-t_0/\overline{\tau_{\mathrm{off}}}})}{1-(1-e^{-t_0/\overline{\tau_{\mathrm{off}}}})} = 1 - e^{-(t-t_0)/\overline{\tau_{\mathrm{off}}}}, \quad \text{if } t > t_0.$$

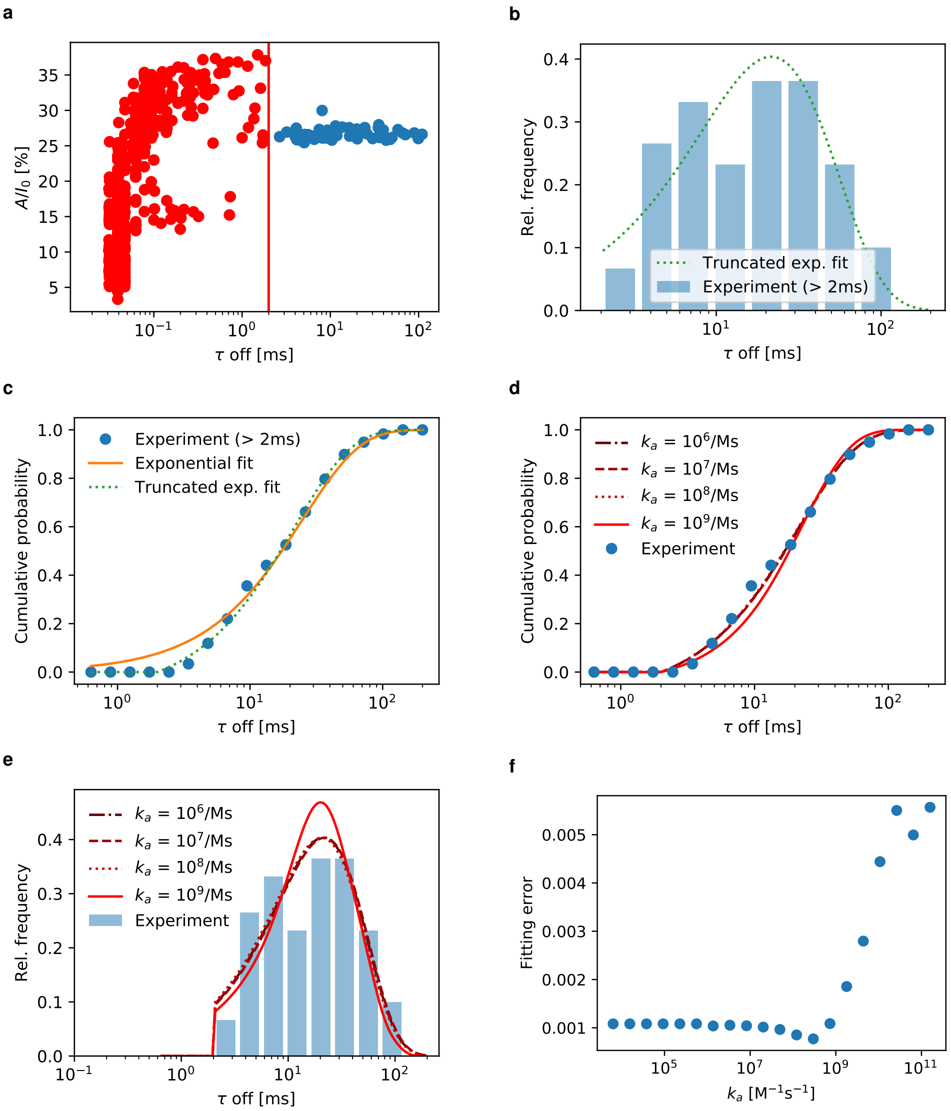

Fig. S6 | Binding of protein trypsin to the DNA nanopore, part I: Long binding. a, Current events recorded for a pore described by Diederichs *et al.*[11] Events colored in red ($\tau_{\text{off}} \leq 2$ ms) are discarded. **b–f**, Results of fitting various statistical models to the data, as explained in the text.

The CDF obtained after truncation at $t_0 = 2$ ms is now plugged into the fitting algorithm to determine the unknown mean time parameter $\overline{\tau_{\text{off}}}$. In Fig. S6c, both the truncated and the non-truncated exponential fits are shown, and we clearly see that the truncated version is superior in capturing the data distribution in the lower range. The PDF (probability density function) of the truncated fit is plotted in Fig. S6b alongside the data histogram. We note that, visually, agreement for the PDF seems to be worse than for the CDF. This is because the empirical CDF is basically the integrated histogram and therefore, in general, much smoother, as stochastic fluctuations are averaged out by integration. For small datasets such as this one, it is therefore preferable to use the CDF for model fitting (since we don't want to fit to stochastic noise).

Our actual binding model involves Poisson-guided binding and re-binding during the BD simulation to a section of the nanopore (Fig. 5c, main text). It produces stochastic dwell times in a way that is more complicated than simply drawing exponential variables. Still, it meets the requirement for applying our fitting algorithm: the CDF can be evaluated

efficiently. To accomplish this, our BD-binding algorithm, instead of generating dwell times directly, is modified to only record the *number* of bindings for each event. Events with no binding are discarded. Conditioned on a given number of bindings $n \geq 1$, the dwell time—being the sum of n exponentially distributed binding times—follows a Gamma distribution with CDF

$$F_n(t;k_d) := \frac{\gamma(n,k_d t)}{(n-1)!}.$$

where $\gamma(\cdot,\cdot)$ is the incomplete gamma gunction. This CDF directly depends on one of the model parameters, the dissociation rate k_d. The final, unconditional CDF is computed by averaging over 1000 independent draws of n,

$$F(t;k_d,k_a) := \overline{F_n(t;k_d)}.$$

The second parameter, the association rate k_a, enters *via* its influence on the distribution of n. In the limit where $k_a \to 0$, this model would reduce to the exponential distribution above (with $\overline{\tau_{\text{off}}} = k_d^{-1}$). Evaluations of $F(t;k_d,k_a)$ are implemented efficiently by precomputing and reusing a fixed number of trajectories, as explained in the Methods. To fit the cut-off distribution of long current events, we again use the modification of Eq. (3) to obtain a truncated CDF.

For reasons which will be apparent immediately, fitting k_a directly is not robust, *i.e.* it does not yield a reliable and reproducible result. Therefore our approach is to fix k_a and, for each fixed k_a, perform a fit of k_d. Resulting CDFs and PDFs are shown in Figures S6d and S6e, respectively. For displayed values $k_a \leq 10^8\,\text{M}^{-1}\text{s}^{-1}$, the distributions are indistinguishable from each other; only $k_a = 10^9\,\text{M}^{-1}\text{s}^{-1}$ looks significantly different and yields a worse fit to the data. The fit quality is assessed in Fig. S6f, which shows the minimal value of the cost function from Eq. (2), normalized as $\frac{1}{N+1}L(\theta)$, for different k_a. The slight kink in the error surface near $k_a = 5 \cdot 10^8\,\text{M}^{-1}\text{s}^{-1}$ does not allow a conclusion about its value, as such a small fluctuation in the fitting error can easily result from stochasticity in the data. The only valid conclusion here is that the association rate can have any value smaller than $10^9\,\text{M}^{-1}\text{s}^{-1}$.

In the simulations shown in the main text, we have (arbitrarily) set k_a to $10^8\,\text{M}^{-1}\text{s}^{-1}$, which leads to 0.78 bindings per event on average. Our fitting procedure then yields $k_d = 67\,\text{s}^{-1}$, corresponding to an average binding duration of $1/k_d = 15\,\text{ms}$.

Short and long binding The next step is to model current event durations over the entire observable time range. Because the measurement device used in the experiments filtered events at 10 kHz, we discard all events shorter than $100\,\mu\text{s} = (10\,\text{kHz})^{-1}$ to avoid dealing with artificial distortions in the distribution; see Fig. S7a. This time, the empirical CDF of the data does not resemble an exponential distribution at all—see Fig. S7b. A better model is given by the *double* exponential distribution

$$F(t;\tau_1,\tau_2,w) = 1 - \frac{w}{1+w}e^{-t/\tau_1} - \frac{1}{1+w}e^{-t/\tau_2}. \tag{4}$$

It has three parameters—the two characteristic times τ_1 and τ_2 and the weight w—and models two independent exponential binding processes, where the first one is w times as likely as the second one. For Fig. S7b, we truncate this CDF at $t_0 = 100\,\mu\text{s}$ like the data, using Eq. (3); see also Fig. S7c for the PDF and histogram.

As in the case of the single binding process, we want to estimate the parameters of our full binding model, which this time involves binding to two different sections of the pore (Fig. 5d, main text) and the estimation of four kinetic binding parameters: k_a, k_d, $k_{a,2}$ and $k_{d,2}$. Proceeding as before, we can obtain *two* numbers of bindings from our simulation, n_1 and n_2, one for each type of binding. This time, we exclude events where *both* n_1 and

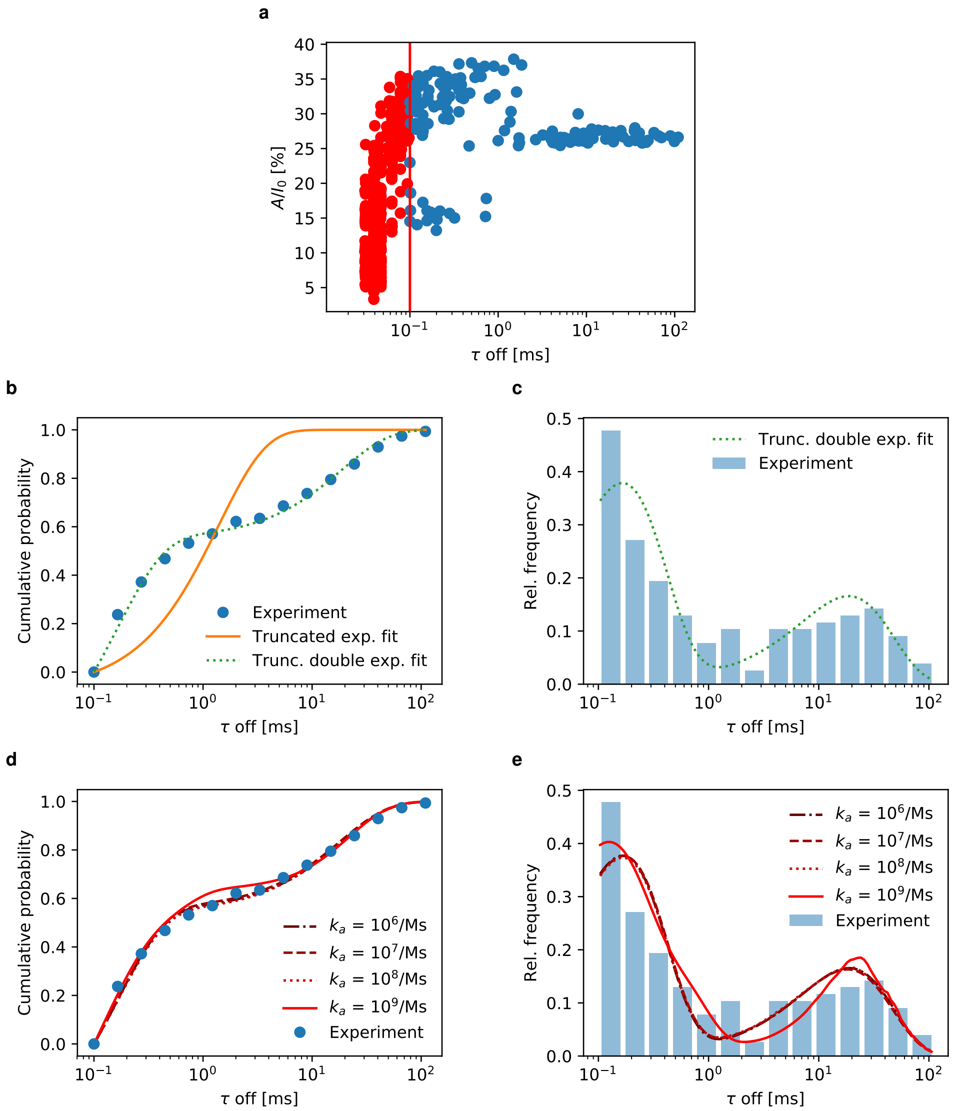

Fig. S7 | Binding of protein trypsin to the DNA nanopore, part II: Short and long binding. a, Current events recorded for a pore described by Diederichs *et al.*[11] Events colored in red ($\tau_{\text{off}} \leq 100\,\mu$s) are discarded. **b–e**, Results of fitting various statistical models to the data, as explained in the text.

n_2 are zero. The distribution of dwell times conditioned on n_1, n_2 is a sum of two gamma distributions $T_1 + T_2$ with different shape and scale parameters, whose CDF admits no simple closed-form. However, if we assume that the second binding process is much faster than the first one, $T_2 \ll T_1$, we can approximate

$$T_1 + T_2 \approx \begin{cases} T_1 & \text{if } n_1 > 0, \\ T_2 & \text{if } n_1 = 0 \text{ and } n_2 > 0. \end{cases}$$

Thus, in this appproximation, the unconditional distribution of $T_1 + T_2$ reduces to a simple superposition of the distributions of T_1 and T_2, weighted by their respective probabilities, p_1 and $(1 - p_1)p_2$, where $p_i = P(n_i > 0)$. The final, unconditional CDF is

$$F(t; k_d, k_{d,2}, k_a, k_{a,2}) := \frac{w}{1+w}\overline{F_{n_1}(t; k_d)} + \frac{1}{1+w}\overline{F_{n_2}(t; k_{d,2})}, \tag{5}$$

with relative weight $w = \frac{p_1}{(1-p_1)p_2}$. The first average is taken over events where $n_1 > 0$, the second over events where $n_1 = 0$ but $n_2 > 0$; both p_1 and p_2 are evaluated empirically. In the limit where k_a and $k_{a,2}$ both tend to zero while keeping w constant, we would recover the double exponential distribution (4). (Actually, in this limit, $w = \frac{p_1}{p_2}$ and we do not even need the approximation $T_2 \ll T_1$, because binding of the two different types will never occur at the same time, so either n_1 or n_2 will always be zero).

Similar to before, we fix the association rate constant k_a at different values and estimate the remaining parameters by plugging the truncated version of our CDF (5) in our fitting algorithm above. Figures S7d and S7e display the results. For any given k_a, the second association rate constant $k_{a,2}$ is well encoded in the relative weight w of the two gamma-type distributions, and the fitting procedure reliably arrives at the same value of $k_{a,2}$ in multiple different trials. Again, only an upper bound can be given for k_a. Fixing $k_a = 10^8\,\mathrm{M}^{-1}\mathrm{s}^{-1}$ while fitting all remaining parameters leads to the estimates $k_d = 77\,\mathrm{s}^{-1}$, $k_{d,2} = 6434\,\mathrm{s}^{-1}$ and $k_{a,2} = 6.5 \cdot 10^6\,\mathrm{M}^{-1}\mathrm{s}^{-1}$. In particular, the estimated value of the slow dissociation rate k_d ($77\,\mathrm{s}^{-1}$) is close to the value obtained for a single type of binding ($67\,\mathrm{s}^{-1}$).

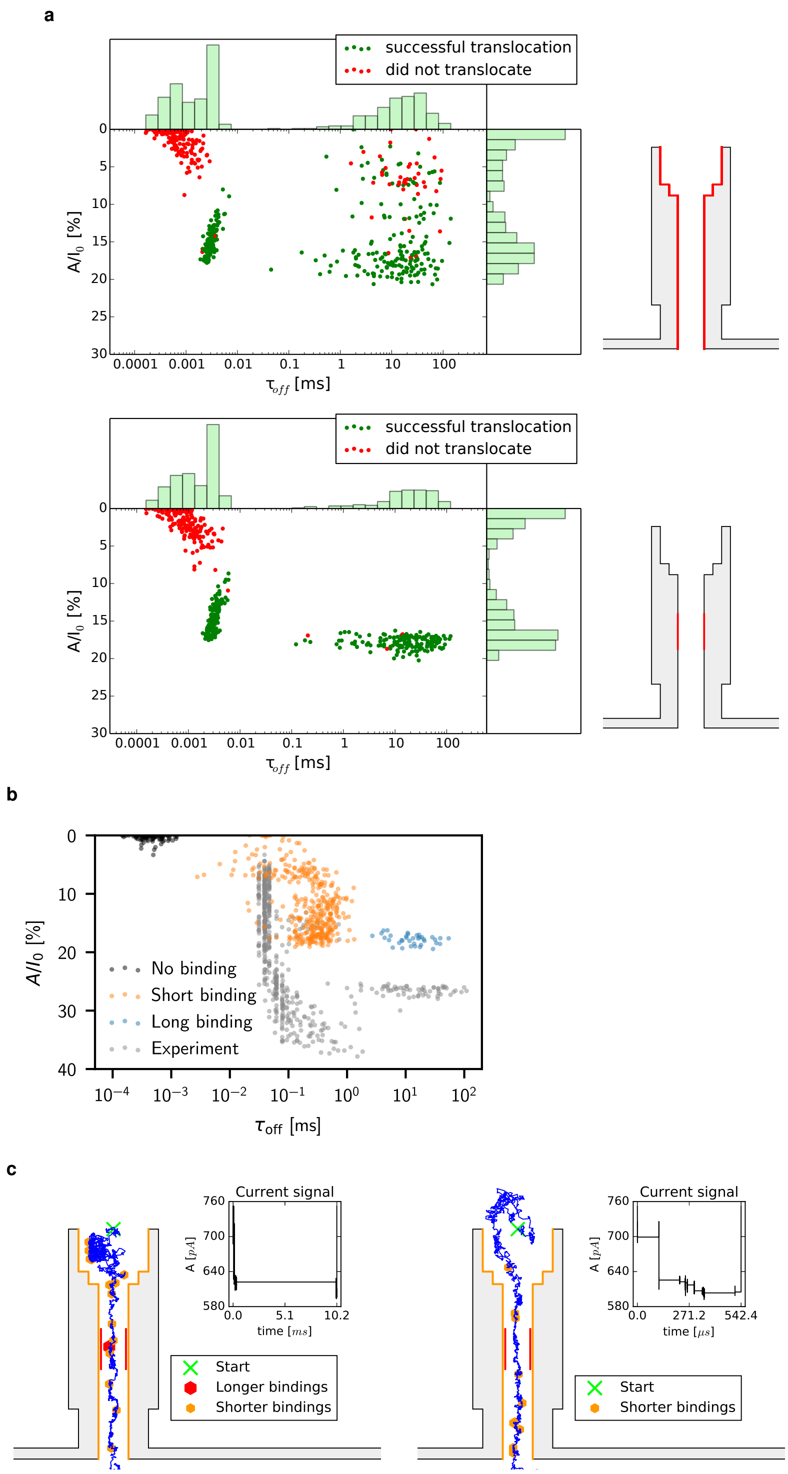

Fig. S8 | Simulation of unspecific pore-protein interaction in DNA nanopore. a, Simulated events with a single type of unspecific binding in different parts of the nanopores. **b**, Version of Figure 5d (main text) with alternative coloring which indicates the type of binding. **c**, Trajectories and current traces with two different types of binding.

Notes and references

[1] Bhattacharya, S.; Muzard, J.; Payet, L.; Mathé, J.; Bockelmann, U.; Aksimentiev, A.; Viasnoff, V. Rectification of the Current in Alpha-Hemolysin Pore Depends on the Cation Type: The Alkali Series Probed by Molecular Dynamics Simulations and Experiments. *J. Phys. Chem. C* **2011**, *115*, 4255–4264.

[2] Simakov, N. A.; Kurnikova, M. G. Soft Wall Ion Channel in Continuum Representation with Application to Modeling Ion Currents in Alpha-Hemolysin. *J. Phys. Chem. B* **2010**, *114*, 15180–15190.

[3] Makarov, V. A.; Feig, M.; Andrews, B. K.; Pettitt, B. M. Diffusion of Solvent around Biomolecular Solutes: A Molecular Dynamics Simulation Study. *Biophys. J.* **1998**, *75*, 150–158.

[4] Happel, J.; Brenner, H. *Low Reynolds Number Hydrodynamics: With Special Applications to Particulate Media*; Martinus Nijhoff: The Hague, 1983.

[5] Dettmer, S. L.; Pagliara, S.; Misiunas, K.; Keyser, U. F. Anisotropic Diffusion of Spherical Particles in Closely Confining Microchannels. *Phys. Rev. E - Stat. Nonlinear, Soft Matter Phys.* **2014**, *89*, 062305.

[6] Wei, R.; Gatterdam, V.; Wieneke, R.; Tampé, R.; Rant, U. Stochastic Sensing of Proteins with Receptor-Modified Solid-State Nanopores. *Nat. Nanotechnol.* **2012**, *7*, 257–263.

[7] Lata, S.; Reichel, A.; Brock, R.; Tampé, R.; Piehler, J. High-Affinity Adaptors for Switchable Recognition of Histidine-Tagged Proteins. *J. Am. Chem. Soc.* **2005**, *127*, 10205–10215.

[8] Smoluchowski, M. v. Versuch einer mathematischen Theorie der Koagulationskinetik kolloider Lösungen. *Z. Phys. Chem.* **1917**, *92*, 129.

[9] Schreiber, G.; Haran, G.; Zhou, H.-X. Fundamental Aspects of Protein-Protein Association Kinetics. *Chem. Rev.* **2009**, *109*, 839–860.

[10] Plesa, C.; Kowalczyk, S. W.; Zinsmeester, R.; Grosberg, A. Y.; Rabin, Y.; Dekker, C. Fast Translocation of Proteins through Solid State Nanopores. *Nano Lett.* **2013**, *13*, 658–663.

[11] Diederichs, T.; Pugh, G.; Dorey, A.; Xing, Y.; Burns, J. R.; Nguyen, Q. H.; Tornow, M.; Tampé, R.; Howorka, S. Synthetic Protein-Conductive Membrane Nanopores Built with DNA. *Nat. Commun.* **2019**, *10*, 5018.
